# Supplementary material for: Cooperative mechanisms of oxide ion conduction in tellurites with secondary bond interactions and Grotthuss-like processes
Source: Nat Commun. 2025 Feb 4;16:1353. doi: 10.1038/s41467-025-56108-1 (PMC11794846; doi:10.1038/s41467-025-56108-1)
Supplement: Supplementary file 1 — Supplementary information [file 41467_2025_56108_MOESM1_ESM.pdf]

Supplementary Information

Cooperative Mechanisms of Oxide Ion Conduction in Tellurites with Secondary Bond Interactions and Grotthuss-like Processes

Zhenyu Zhu#[1], Guanqun Cai#*[1, 2], Yuxiang Feng[1], Juping Xu[3, 4], Shengqi Chu[5], Pengfei An[5], Jianrong Zeng[6, 7], Wen Yin[3, 4], Yu Gu*[8], Xiaojun Kuang*[9] and Junliang Sun*[1]

[1]College of Chemistry and Molecular Engineering, Beijing National Laboratory for Molecular Sciences, Peking University, Beijing, 100871, P.R. China

[2]Institute of Atomic and Molecular Physics, Sichuan University, Chengdu, 610065, P.R. China

[3]Institute of High Energy Physics, Chinese Academy of Sciences, Beijing, 100049, P.R. China

[4]Spallation Neutron Source Science Center, Dongguan, 523803, P.R. China

[5]Beijing Synchrotron Radiation Facility, Institute of High Energy Physics, Chinese Academy of Sciences, Beijing 100049, P.R. China

[6]Shanghai Synchrotron Radiation Facility, Shanghai Advanced Research Institute, Chinese Academy of Sciences, Shanghai, 201204, P. R. China

[7]Shanghai Institute of Applied Physics, Chinese Academy of Sciences, Shanghai, 201800, P. R. China

[8]College of Physics and Optoelectronic Engineering, Jinan University, Guangzhou, 510632, P. R. China

[9]Guangxi Key Laboratory of Electrochemical and Magnetochemical Functional Materials, College of Chemistry and Bioengineering, Guilin University of Technology, Guilin, 541004, P. R. China

# These authors contributed equally to this work

* Email: junliang.sun@pku.edu.cn; guanqun.cai@pku.edu.cn; kuangxj@glut.edu.cn; tygu@jnu.edu.cn;

Supplementary Note 1. Similarities between secondary bonds and hydrogen bonds

The term of secondary bond was established by Alcock[1] to name a class of noncovalent interaction between main-group element and one or more atoms which typically possess lone pairs of electrons. SBIs are stabilized by orbital, electrostatic and dispersion contributions[2,3]. In Y−A···X, The σ^*(A−Y) molecular orbital overlap with a non-bonding p-orbital from X[3]. Meanwhile, the electrostatic contribution could add to the directional nature of the SBI via σ-hole bonding[4]. The nature of this interaction derived from empirical observation have been corroborated by modern quantum mechanical methods[1–3,5,6]. Since the electronegativity of atoms decreases and polarizability increases down the periodic table, the difference of orbital energy between the σ(A−Y) and σ^*(A−Y) decreases and the strength of the dispersion force increases, resulting in stronger SBIs for tellurium compared to selenium and sulfur[3,7]. According to the above introduction, the form and fundamental of SBIs are close to H-bond interactions[8–10]. Further, B. Silvi *et al.* consider H-bonds are involved in an unified chemical model of secondary bonding[11].

Meanwhile, SBIs in Te system have comparable or even higher strengths and relative stabilities against hydrogen bonds generally known to be the strongest of non-covalent interactions[5,6,12,13]. In fact, a number of supramolecular structures assembled via tellurium-centred SBIs, similar to H-bond supermolecules, have been reviewed according to L. Haiduc[14] and A. F. Cozzolino[3]. M. K. Si *et al.* computed the Te···O interaction energies in quasi-cyclic systems with the M06-2X/aug-cc-pVQZ level of DFT theory. The DFT calculated results suggest that the H−Te···O interaction energy is 27.7 kJ mol^{-1} with 2.67 Å distance and fluorine substituted F−Te···O interaction energy is 99.5 kJ mol^{-1} with 2.16 Å distance, which are comparable or stronger than the hydrogen bond energies in similar systems[5]. Cozzolino *et al.* calculated N−Te···N interaction energy to be 31.5 kJ mol^{-1} with 2.77 Å distance in telluradiazole dimer[6].

The Grotthuss mechanism through hydrogen-bonding networks can be found in in aqueous systems[15], ice[16], polymers[17], proteins[18], hydrated metal-organic frameworks[19], covalent organic frameworks[20] and so on. Similarly, secondary bonds can interconnect [TeO_3] or other polyhedra to form continuous networks in crystal structure inorganic compounds akin to hydrogen bond networks in above mentioned systems[21].

Supplementary Note 2. Coordination of secondary bonds

Although the interatomic distances are difficult to be used as an accurate measure of actual strength of interactions alone, it can serve as semiquantitative indicators of relative strength. The Te-O distances distribution was shown in Supplementary Fig. 8a from the average structure model of $Bi_2Te_2O_7$ and $Bi_2Te_4O_{11}$. The bond length is actually concentrated in the four ranges of 1.84-2.07 Å, 2.28-2.47 Å, 2.63-3.01 Å, and 3.19-3.50 Å. This result is consistent with the distribution of Te-O bond lengths in 19 tellurite crystal structures analyzed by Zemann[22]. Based on the distribution, the first three ranges were corresponded to covalent bonds, short and secondary bonds. In addition, the peak of Te-O distance is also obviously discrete in the supercell structure model obtained by RMC, and its local minimum value is about 2.1 Å and 2.6 Å (Supplementary Fig. 8b).

Bond angles with the corresponding covalent bond were necessary be considered when identifying secondary bonds, which >165° generally. The bond lengths and angles of all Te-O bonds with distances less than 3.5 Å in $Bi_2Te_2O_7$ and $Bi_2Te_4O_{11}$ are shown in the Table SX. In the distance

range of 2.2-2.6 Å, all bond angles are greater than 165°. Within the range of 2.6-3.1 Å, a few bond angles are less than 160° around 3 Å, thereby failing to meet the bond angle criteria for secondary bonds, and other O−Te···O angles are >165°, which are long secondary bonds. For the Te-O bonds with distance >3.1 Å, all corresponding bond angles fall below 160°, indicating the absence of secondary bonds.

Voronoi-Dirichlet approach was three-dimensional geometrical methods of crystallochemical analysis based on the Voronoi-Dirichlet partition of crystal space. Voronoi-Dirichlet polyhedron, consisting of perpendicular bisector planes with neighbouring atoms, reflects the form of atomic domains. In Voronoi-Dirichlet approach, the determination of coordination for neighbouring atoms relied on the solid angles of the polyhedron faces. The solid angle ratio > 10% indicates that the central atom is strongly connected with the neighbouring atoms, while the value < 5% suggests no coordination[23]. The solid angles in Te atomic domains were calculated (Supplementary Table 9-12), revealing that the solid angles of O atoms with a distance greater than 3.1 Å are all below 5%. Moreover, the solid angles of the covalent bonds approximate 20%, while the solid angles of the short and long secondary bonds are about 15% and 10% severally. According to this theory, the cutoff values 2.1 Å, 2.6 Å and 3.1 Å are appropriate.

The rules have been established to explain the local structures for the resulting geometries included SBIs[1]. The first one is that the geometry of the primary bonds of the central atom is governed in the usual way by the number of lone and bonded electron pairs. The second one is describing that secondary bonds can form in any direction in line with primary bonds. however, the last one is that SBIs not in the same direction as a lone pair on the central atom.

In theory, SBIs should weaken the primary bond. The secondary bond might enhance interaction with d orbitals of central atom, without affecting or even strengthened the primary bond in a few cases[1]. In a word, the formation of the secondary bond should change the resulting geometries and symmetries for the Te-O polyhedra. This is the theoretical basis of using the degree of peak splitting corresponding to [TeO3] groups in infrared and Raman spectrum as an indirect indicator for assessing the strength or quantity of secondary bonds in the material.

Supplementary Note 3. RMC simulation and partial atomic pair distribution function

The static average structure analysis help identifying the possible oxygen pathways. However, oxygen conduction is a dynamic process in a structure with high concentrations of defects and disorder. This is exactly where the static average structures are difficult to provide further deeper information. A more detailed investigation of the local structure disorder and dynamics is needed to fully unveil the oxygen conduction in these bismuth tellurites. Moreover, due to the intricate electronic structure of bismuth tellurites and SBIs, conventional simulation methods such as DFT or MD are difficult to accurately reproduce the materials dynamic structures. Therefore, we combined the experimental total scattering method and the reverse Monte Carlo simulation (RMC) method to investigate its mechanism at the atomic structure scale. The refined RMC configuration is a snapshot of the instantaneous atomic structure of the material. In the other words, the refined supercell structure model keeps the periodic structure in an averaged picture while presents the local structure disorder hidden in the diffuse scattering with the refined individual atomic positions.

Supplementary Fig. 19 shows the distances for O-O, Te-O, Bi-O, Te-Te, Bi-Te, Bi-Bi and atom pair distances respectively in $Bi_2Te_4O_{11}$ at the room temperature and 600 °C. The Te-Te distances shift to larger distances which is consistent to the positive thermal expansion behaviour demonstrated in Supplementary Fig. 2. However, the Te-O covalent bonds (first peak in Supplementary Fig. 19b) and the first neighbour O-O distances (first peak in Supplementary Fig. 19a) don't show any expansion from the room temperature to 600 °C except the distribution become broader.

Combining with the Bi-Bi, Bi-Te and Bi-O distances shown in Fig. 2 and Supplementary Fig. 19, it can be concluded that the thermal expansion of $Bi_2Te_4O_{11}$ from RT to 600 °C is mainly ascribed to the expanded heavy atom (Bi, Te) distances. The basic Te-O covalent bonds and Bi-O ionic bonds anomalously remain the same overall average distances though the temperature increased nearly 600 °C. More interestingly, despite the fact that the heavy atom distances expanded and the oxygen atoms connected to them remain the same average distances with them, the oxygen atoms also don't show any expansion. Instead, they become substantially more widely distributed. These results were also verified by Bi-O path length fitted with EXAFS spectra (Supplementary Fig. 20 and Table 13).

It can be inferred from the above first neighbour distance analysis that as temperature increases, while the pivot heavy atoms expand, the Te-O and Bi-O basic units maintain integrity (unchanged average distances) as well as become much more flexible (wider distance distribution). This deduction not only allows the oxygen distances at HT remain similar as RT but also explains the substantial wider O-O distances distribution at HT.

Supplementary Note 4. Oxygen vacancies in $Bi_2Te_2O_7$ and $Bi_2Te_4O_{11}$

Although the oxygen atom position occupancy refined by neutron diffraction show no obvious oxygen vacancy, for the O 1s core level spectra, the oxygen vacancy peaks can be clearly identified. In detail, three typical peaks at around 530.0, 531.5, and 532.9 eV shown in Supplementary Fig. 6 a, b can be attributed to lattice oxygen, oxygen vacancies, and surface-adsorbed oxygen species, respectively[24,25]. The results show that the concentration of oxygen vacancies in $Bi_2Te_2O_7$ is higher than $Bi_2Te_4O_{11}$ as shown in Supplementary Fig. 6a and Table 3. The oxygen vacancies discussed here are relative to the intrinsic structure of $Bi_2Te_2O_7$ and $Bi_2Te_4O_{11}$ rather than the fluorite structure. We suggest that these oxygen vacancies generated through intrinsic thermal defect and low levels of TeO_2 or Bi_2O_3 loss during ceramic processing.

Furthermore, the experimental results also show that even $Bi_{0.198}Ca_{0.02}Te_2CaO_{7-\delta}$ (Supplementary Fig. 28), which introduces oxygen vacancies by Ca^{2+} doping, has lower conductivity than undoped $Bi_2Te_4O_{11}$. It indicates that there was indeed a contradiction in the view of traditional mechanism of oxygen vacancy migration, including the traditional polyhedral coordination mechanism with oxygen vacancies as carriers.

Supplementary Note 5. The contribution of Bi-O sublattices

It is generally accepted that the high polarisability electronic structure of Bi^{3+} ions with $6s^2$ lone pair are crucial to anion migration of many Bi-based oxide-ion conductors[26]. The significance of

cation polarizability in the conduction process has been demonstrated through computer simulations by M. S. Islam[27], D. S. Aidhy[28], et. al. as it can facilitate lattice relaxation and displacements of the cations away from the mobile anions. The hybridization of the Bi $6s^2$ lone pair electrons with the oxygen $2p$ orbitals results in a weak Bi-O covalency[29]. Consequently, this leads to a diverse range of non-cubic coordination polyhedron with varying Bi-O bond lengths (Table S8, S9). Therefore, the high polarisability of Bi-O lattice and weak Bi-O interactions offer flexible pathways that allow Te-O polyhedra rotation and of oxygen ion migration.

Several studies indicate that Bi strongly affects the oxide ion transport also owing to the orientation of its $6s^2$ lone pair[30]. For example, the oxide conductivity difference between α- and δ-Bi_2O_3 was explained by the ordering and disordering Bi lone pair orientation in the crystal lattice. Those lone pairs oriented in different directions assists the migration of the oxygen ion[31]. In $Bi_2Te_4O_{11}$, two proposed Grotthuss pathways -[-Te1···O5-]-$_n$ and -[-Te3···O10-]-$_n$ are parallel to the b axis, which is primary migration direction of oxide ion. The b axis symmetry of $P\ 1\ 2_1/n\ 1$ space group results in the disorder direction of the lone-pair. The 2_1 screw axis can reverse the component of direction of the lone pair electron in the ac plane, and the glide plane reversed the component of direction along the b axis. $Bi_2Te_2O_7$ also has different directional lone pair of Bi with the space group $P\ 2_1/b\ 2/c\ 2_1/n$.

Supplementary Note 6. Oxygen ion conduction in other tellurites

Three potential oxygen ion conducting materials were screened for synthesis and electrical properties testing based on the proposed mechanism aiming to validate the influence of secondary bonds on oxygen ion conductivity. The structures of $ZrTe_3O_8$, $La_2Te_4O_{11}$ and $Bi_2Te_2WO_{10}$ contain abundant secondary bonds and secondary bonds are alternately connected with covalent bonds to form chains or networks (Supplementary Fig. 28a, 28b, 28c). It should be noted that $La_2Te_4O_{11}$ has a similar chemical formula to $Bi_2Te_4O_{11}$, but the structure is completely different. Bi_2TeO_5 was selected as the reference sample, whose structure contains 3+1 coordination Te-O polyhedra. Each pair of polyhedra is interconnected by SBIs without forming an infinite chain or layer of Te-O connections (Supplementary Fig. 28d).

All four tellurites can be successfully synthesized (Supplementary Fig. 28e) according to the reported synthesis methods[32–35]. The conductivity of Bi_2TeO_5 is exhibits a relatively low value, approximately 1.4×10^{-5} S cm^{-1} at 700 °C, which is consistent with previously reported result[36]. The other three materials showed high electrical conductivity (Supplementary Fig. 28f) and significantly conductive behaviour of oxygen ions according to electrode response in different atmospheres. Further, oxide ion transport number of $ZrTe_3O_8$, $La_2Te_4O_{11}$ and $Bi_2Te_2WO_{10}$ measured by electromotive force method were 0.73, 0.59 and 0.92 at 700 °C, 700 °C and 650 °C, respectively. Oxide ion conductivity can be calculated as 1.0×10^{-3}, 1.7×10^{-4}, 9.5×10^{-4} S cm^{-1}, respectively. The obtained results further validate the significance of SBI in the Grotthuss-like process in oxygen ion migration, and provide guidance for the design and exploration of new oxygen ion conducting materials in tellurite.

Supplementary Note 7. First principle phonon calculation

We attempted to do further accurate phonon calculation. More accurate first-principles calculations are performed by using density functional theory within the generalized gradient

approximation (GGA) in the Perdew-Burke-Ernzerhof adapted for solids (PBEsol) form[37] as implemented in the CP2K code[38]. The core-valence interactions are described through Goedecker-Teter-Hutter (GTH) pseudopotentials[39,39]. We use double-zeta basis sets of MOLOPT quality[40]. The plane-wave energy cutoff for the electron density is set to 800 Ry to ensure the convergence of the total energy. The convergence parameter of structural optimization is set as MAX_FORCE $\leq 10^{-5}$ and EPS_SCF $\leq 10^{-8}$. The phonon band structure is generated by using finite displacement method. The Brillouin zones of the unit cells are sampled with 5×4×2 Monkhorst–Pack special k-point meshes for $Bi_2Te_4O_{11}$ bulk. We use Γ-point sampling with the supercells containing 2×2×1 unit cells. The vibrational frequencies and eigenmodes required for the distorted structure are obtained through finite displacements of 0.01 A of fully relaxed atomic positions generated from phonopy[41].

As shown in Fig.S31, although we have taken great effort in DFT phonon calculation, there are still imaginary modes in the calculated dispersion curve. This indicates the complicate bonding property of Te···O secondary bonds (different from pure short range and long-range interaction) makes it very difficult to use current DFT functionals and long range force corrections to accurately study the lattice dynamics of the bismuth tellurite structure network. Therefore we resort to the experimental total scattering method and the reverse Monte Carlo simulation method to investigate its oxygen ion conduction mechanism at the atomic structure scale.

Supplementary Figures

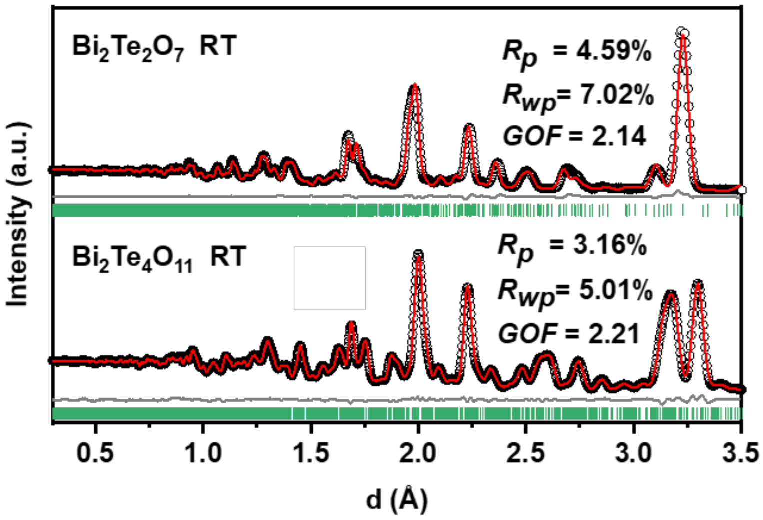

Supplementary Figure 1. Rietveld plot of the neutron pattern of $Bi_2Te_2O_7$ and $Bi_2Te_4O_{11}$ at RT. The observed and calculated intensities and difference plots are shown by black marks, red and gray solid lines, respectively. Green tick marks stand for calculated Bragg peak positions.

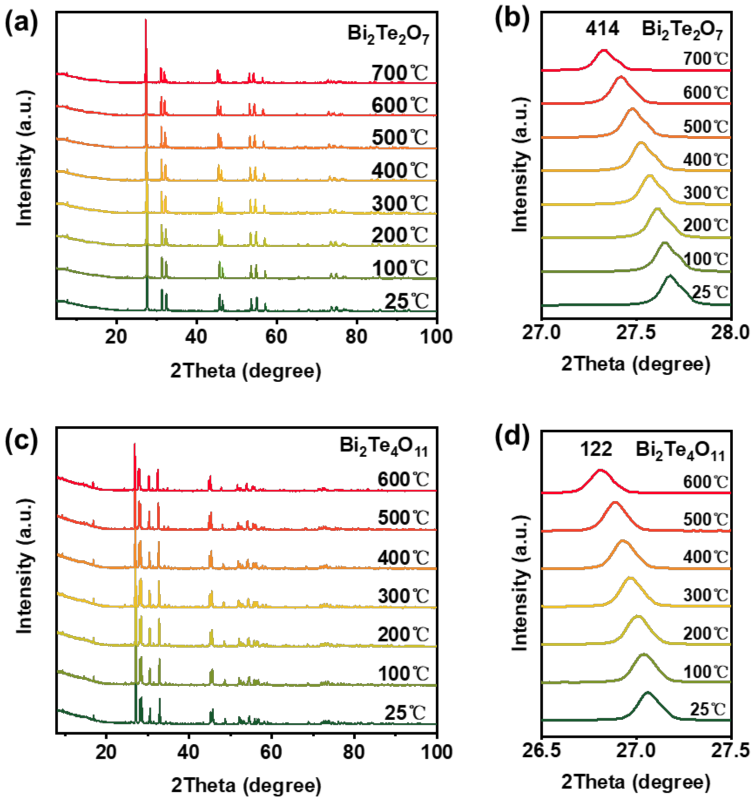

Supplementary Figure 2. VT-XRD patterns (a, c) the VT-XRD patterns of $Bi_2Te_2O_7$ and $Bi_2Te_4O_{11}$ in the temperature range of RT-700 °C and RT-600 °C, respectively. (b, d) Position evolutions of the enlarged VTXRD plots of the 411 reflections in $Bi_2Te_2O_7$ and the 122 reflections in $Bi_2Te_4O_{11}$.

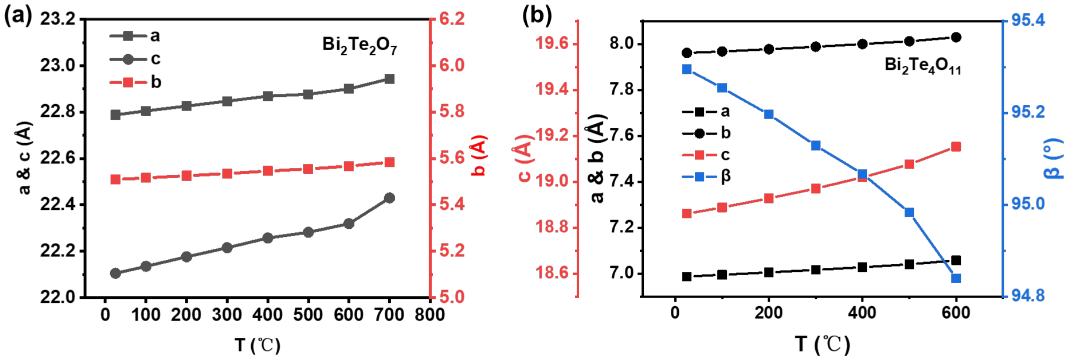

Supplementary Figure 3. Thermal expansion behaviors of the refined cell parameters of $Bi_2Te_2O_7$ and $Bi_2Te_4O_{11}$ with VTXRD. The lattice parameters increase with an increase in temperature. The average thermal expansion coefficient of $Bi_2Te_4O_{11}$ along the c axis is 2.689(2) × 10^{-5} K^{-1} in the temperature range 25-600 °C, which is 1.543 times higher than that along the *a* axis (1.7422(8) × 10^{-5} K^{-1}) and 1.800 times higher than b axis (1.4942(12) × 10^{-5} K^{-1}). The average thermal expansion coefficient of $Bi_2Te_2O_7$ along the c axis is 2.172(2) × 10^{-5} K^{-1} in the temperature range 25-700 °C, which is 2.153 times higher than a axis (1.009(2) × 10^{-5} K^{-1}) that along the and 1.091 times higher than b axis (1.9909(6) × 10^{-5} K^{-1}, indicating a anisotropy in thermal expansion.

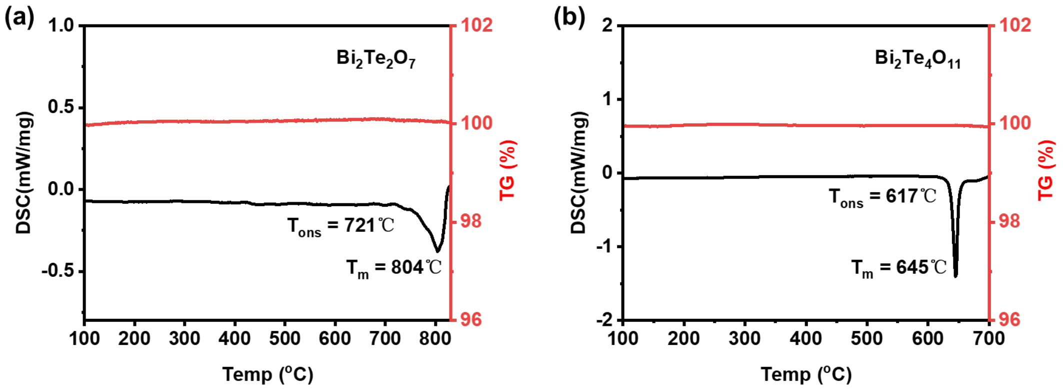

Supplementary Figure 4. Thermogravimetric (TG) and calorimetric evolution (DSC) data of $Bi_2Te_2O_7$ and $Bi_2Te_4O_{11}$. The endothermic peak in the DSC signal corresponds to the melting process. Onset temperature and melting temperature are shown. The melting peak of $Bi_2Te_2O_7$ exhibits onset temperature at 721 °C and the endotherm temperature at 804 °C. $Bi_2Te_4O_{11}$ melts at 645 °C peak temperature and the melting process starts at about 617 °C.

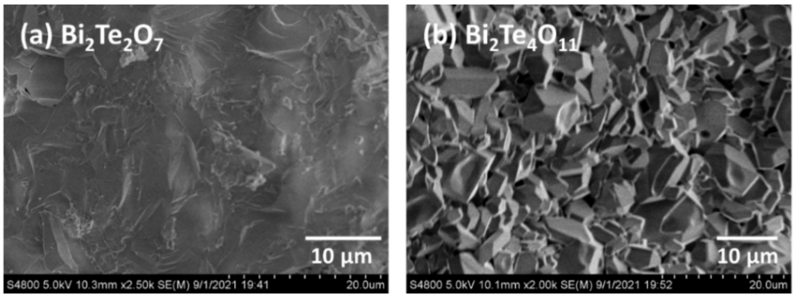

Supplementary Figure 5. SEM internal section micrograph for $Bi_2Te_2O_7$ and $Bi_2Te_4O_{11}$ pellet. The average particle size for $Bi_2Te_2O_7$ and $Bi_2Te_4O_{11}$ is 3-10 μm. The morphology and structure of $Bi_2Te_2O_7$ and $Bi_2Te_4O_{11}$ were investigated by scanning electron microscopy (**SEM**). We succeeded in sintering both of them to dense, single-phase ceramics with minimal porosity and an average grain size around 3-10 mm. There is no trace of liquid phase formation at the grain boundaries. **Density** of $Bi_2Te_2O_7$ is 7.09 g cm^{-3} (> 94 % of theoretical density) and density of $Bi_2Te_4O_{11}$ is 6.54 g cm^{-3} (> 93 % of theoretical density) measured by Archimedes' principle with deionized water as immersion medium.

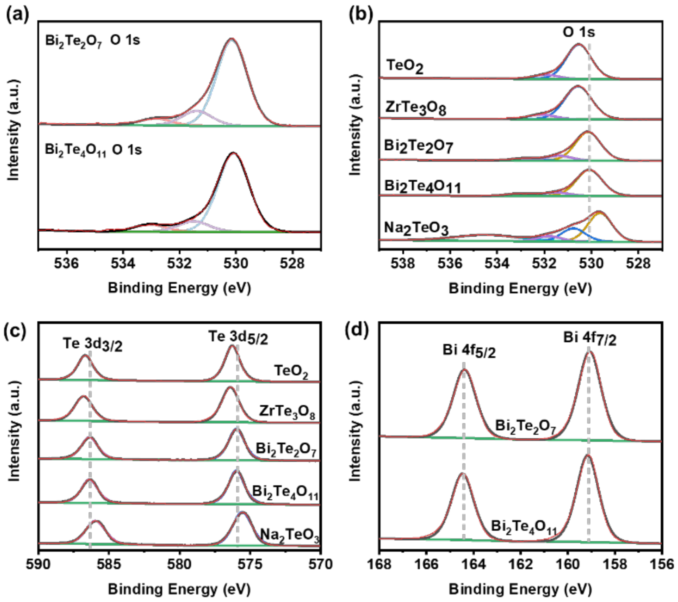

Supplementary Figure 6. XPS spectra. XPS spectra and fit of (a, b) O 1s, (b) Te 3d and (c) Bi 4f in $Bi_2Te_2O_7$, $Bi_2Te_4O_{11}$ and other Te compounds. For each panel, black, red and green solid line represent observed intensities, fitted curve and background, respectively.

The Te $3d_{3/2}$ and $3d_{5/2}$ peak of the tellurium dioxide and tellurite is fitted with two contributions at 586.7-586.0 and 576.3-575.6 eV (SI Fig. Table.). These binding energies (BEs) are related to the presence from $[TeO_4]$ to $[TeO_3]$ units, respectively[42–44]. The Bi spectra have almost no difference between $Bi_2Te_2O_7$ and $Bi_2Te_4O_{11}$ fitted with two peaks at BEs of around 164.4 eV and 159.1 eV corresponding to $4f_{5/2}$ and $4f_{7/2}$ electron energy levels[45].

The O1s photoelectron lines of the investigated compounds are characterized by peaks from 530.5 eV to 529.6 eV (Fig. 9a, b) associated with the transition from [TeO4] to [TeO3][42,44,46]. In addition, the origin of shoulders observed at 531.8-530.9 eV is due to defect sites with low oxygen coordination[24,25]. The higher BEs can be attributed to surface-adsorbed oxygen species[47]. The broad peaks about 535 eV in Na_2TeO_3 are the KLL Auger lines of Na.

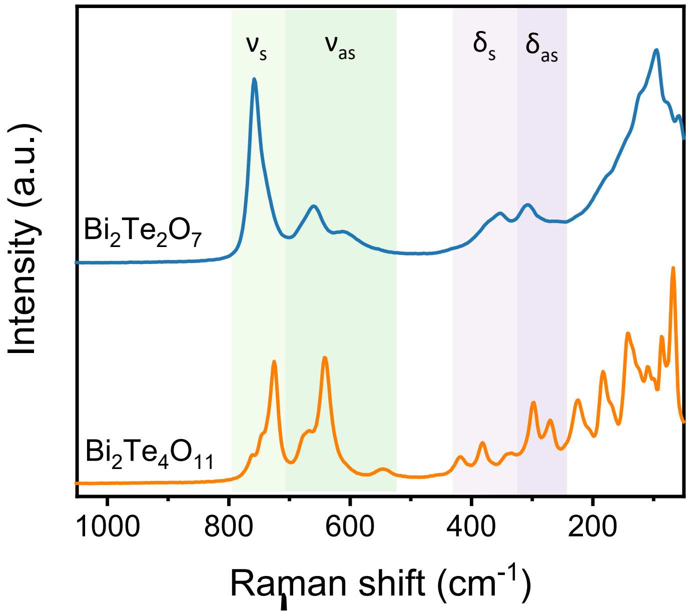

Supplementary Figure 7. **Raman spectrum of $Bi_2Te_2O_7$ and $Bi_2Te_4O_{11}$**

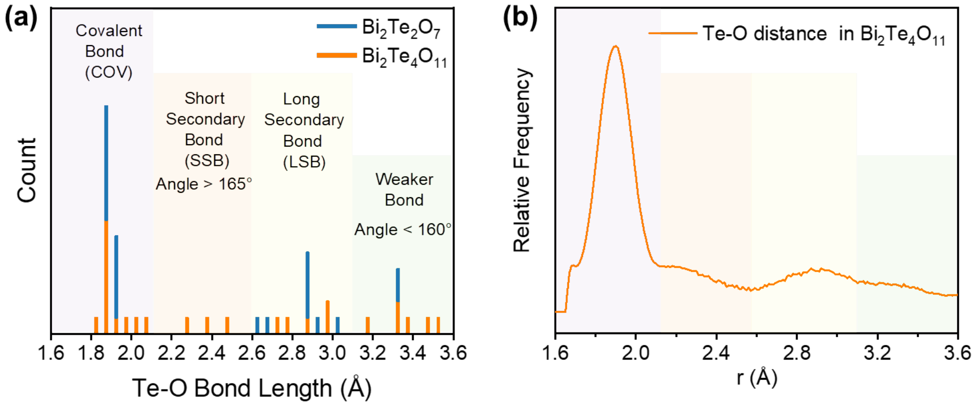

Supplementary Figure 8. **Analysis of Te-O distances.** (a) Histograms for the distribution of Te-O distances in average structure model. (b) Te-O atom pairs distance distribution in $Bi_2Te_4O_{11}$ from 10 RMC configurations.

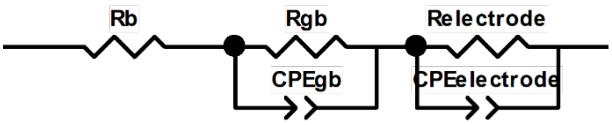

Supplementary Figure 9. **The equivalent circuit model used to fit the impedance data.** The model allowed the deconvolution of the bulk, grain boundary conductivities in total and the calculation of the capacitance values for each of the responses. R and CPE denote a resistor and a constant phase element, respectively, where the subscripts b and gb stand for the bulk and grain boundary, respectively. With increasing temperature, grain boundary response arcs gradually disappear. Therefore, the intercept of the electrode response arc on the Z' axis at high frequency can be estimated as the bulk resistivity, because R_{gb} is much less than R_b and $R_{gb} + R_b \approx R_b$.

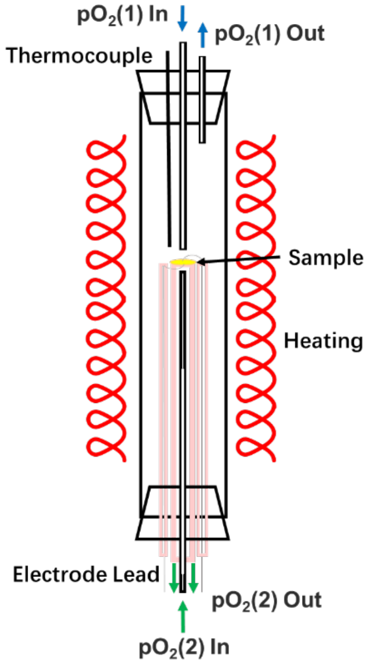

Supplementary Figure 10. Schematic diagram of the devices using for oxygen concentration cell measurement.

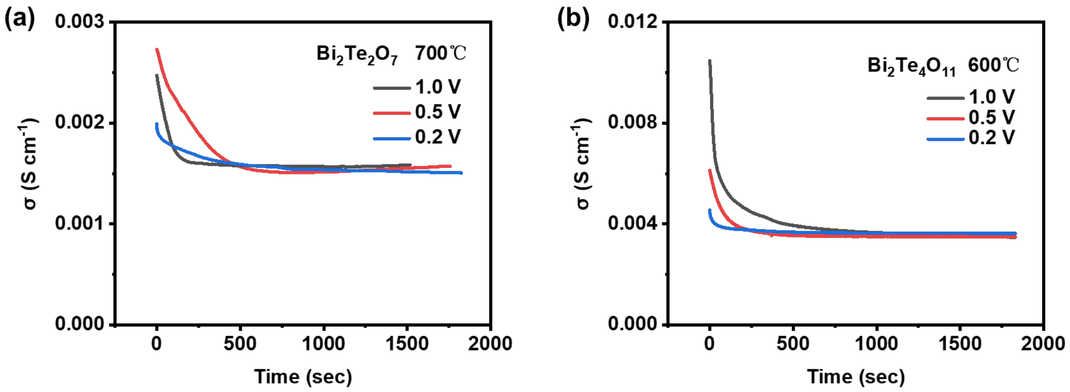

Supplementary Figure 11. Direct current conductivities relaxation curves for 1.0, 0.5 and 0.2 V constant voltage in (a) $Bi_2Te_2O_7$ at 700 °C and (b) $Bi_2Te_4O_{11}$ at 600 °C under N_2 atmosphere.

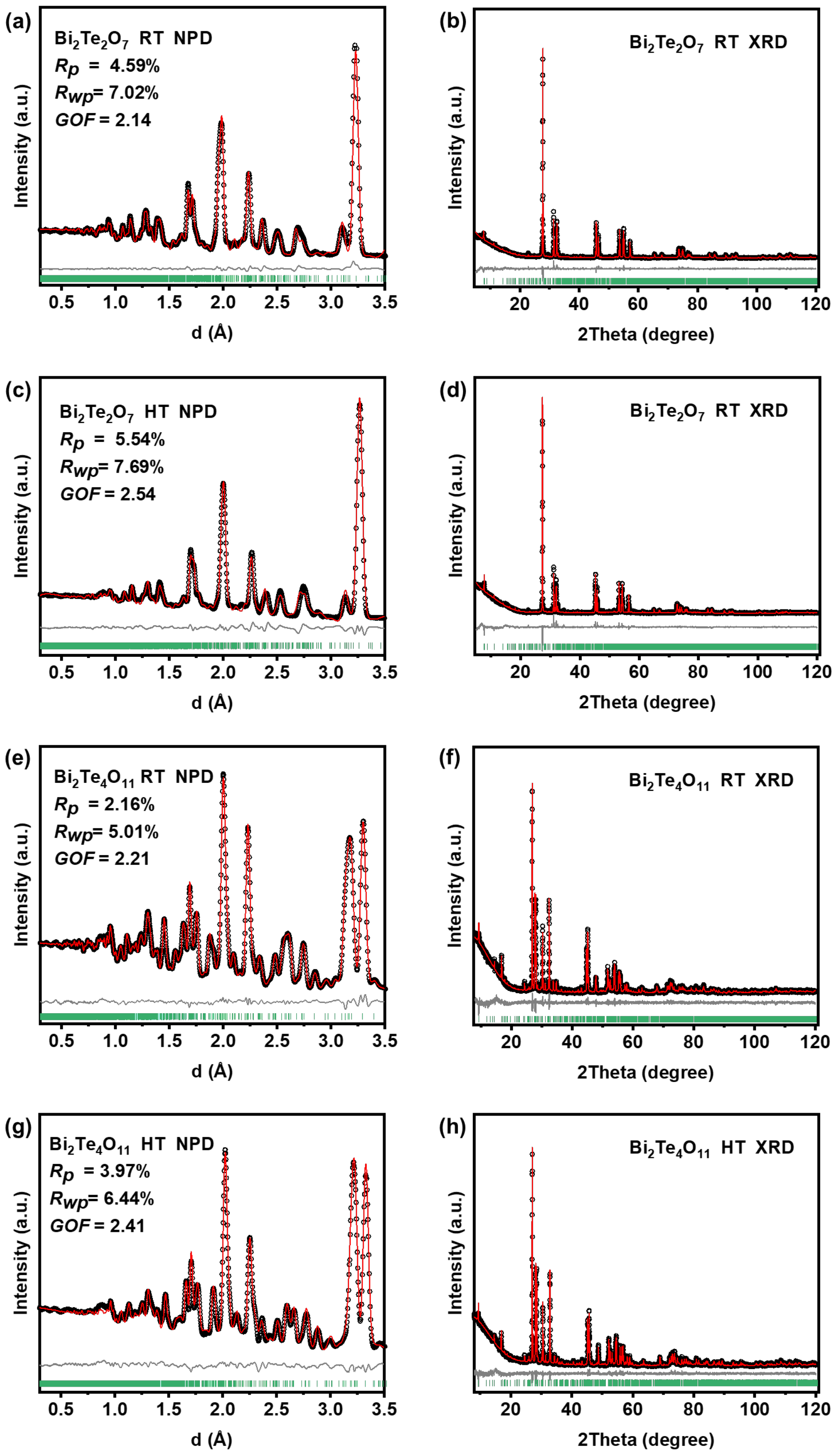

Supplementary Figure 12. Rietveld refinement results of the combined NPD and XRD data. Rietveld refinement against the combined NPD and XRD data for (a, b) $Bi_2Te_2O_7$ at room temperature, (c, d) $Bi_2Te_2O_7$ at 700 °C, (e, f) $Bi_2Te_4O_{11}$ at room temperature and (g, h) $Bi_2Te_4O_{11}$ at 600 °C. The observed and calculated intensities and difference plots are shown by black marks, red and gray solid lines, respectively. Green tick marks stand for calculated Bragg peak positions.

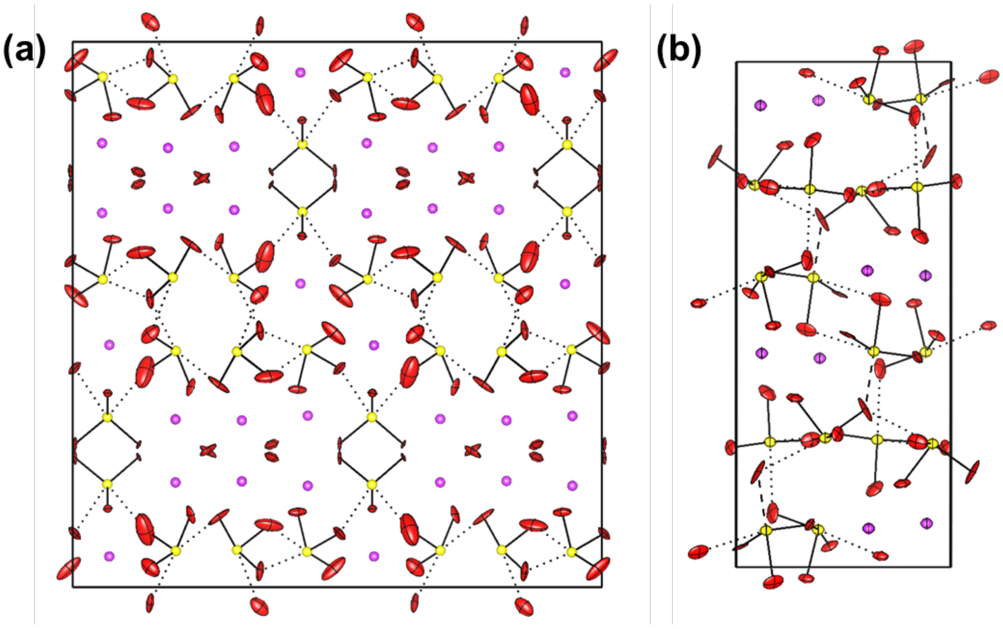

Supplementary Figure 13. Refined crystal structure of $Bi_2Te_2O_7$ and $Bi_2Te_4O_{11}$ with displacement ellipsoids. Refined crystal structure of (a) $Bi_2Te_2O_7$ viewed along the *b*-axis and (d) $Bi_2Te_4O_{11}$ viewed along the *a*-axis at 25 °C with displacement ellipsoids.

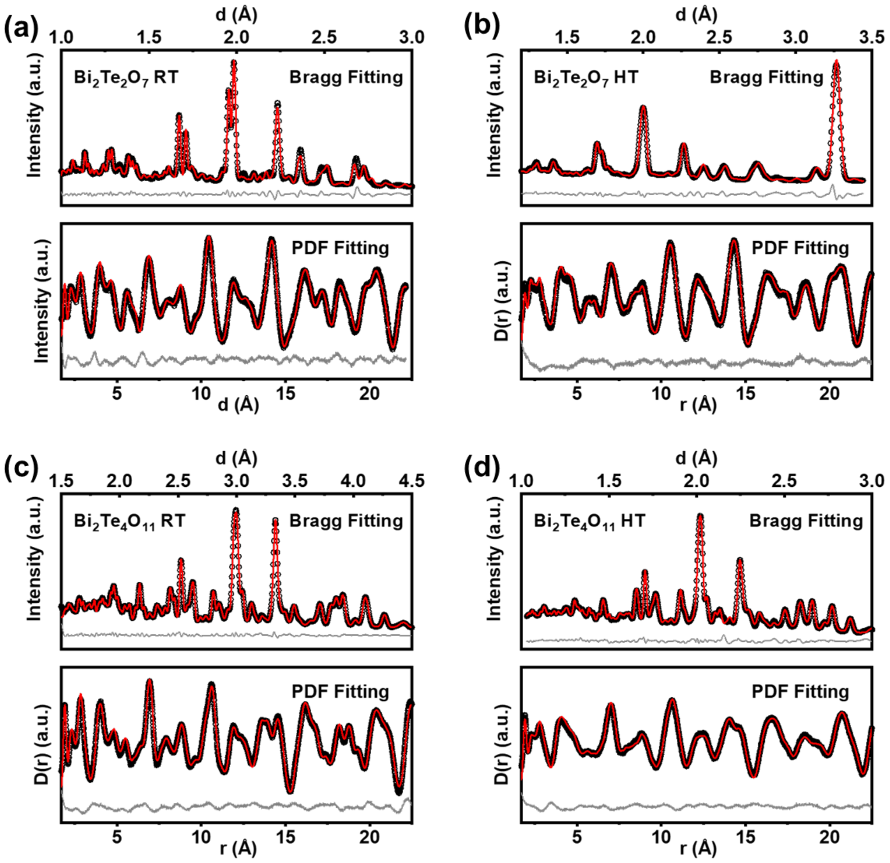

Supplementary Figure 14. RMC fitting results. RMC fitting of the Bragg profile and real space pair distribution function of (a) $Bi_2Te_2O_7$ at 25 °C, (b) $Bi_2Te_2O_7$ at 700 °C and (c) $Bi_2Te_4O_{11}$ at 25 °C, (d) $Bi_2Te_4O_{11}$ at 600 °C.

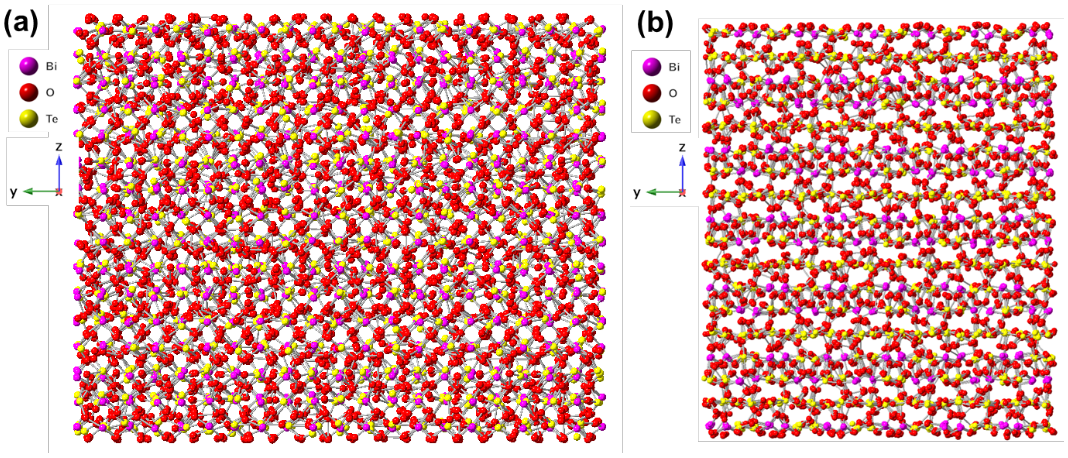

Supplementary Figure 15. RMC refined configurations. RMC refined configurations of (a) 2 × 10 × 2 supercell structure of $Bi_2Te_2O_7$ at 700 °C and (b) 7 × 6 × 3 supercell structure of $Bi_2Te_4O_{11}$ at 600 °C.

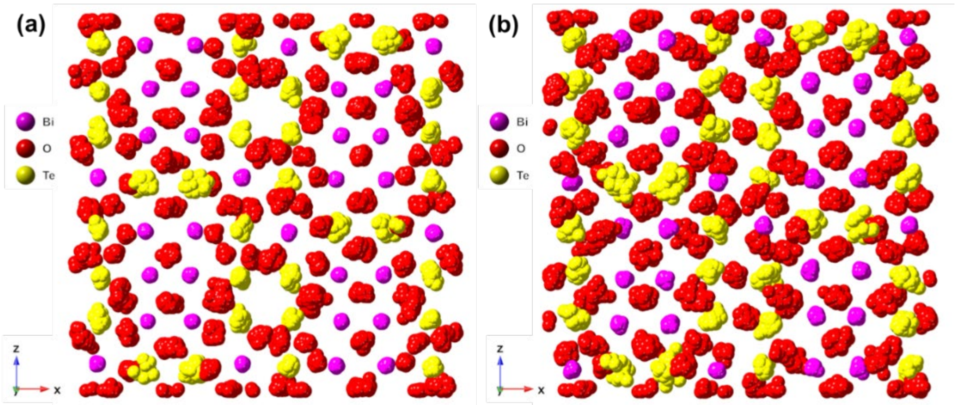

Supplementary Figure 16. The collapsed RMC refined atomic configurations. The collapsed RMC refined atomic configurations of $Bi_2Te_2O_7$ at 25°C and 700 °C, representing its instantaneous of 2 × 10 × 2 supercell structure collapsed into one unit cell.

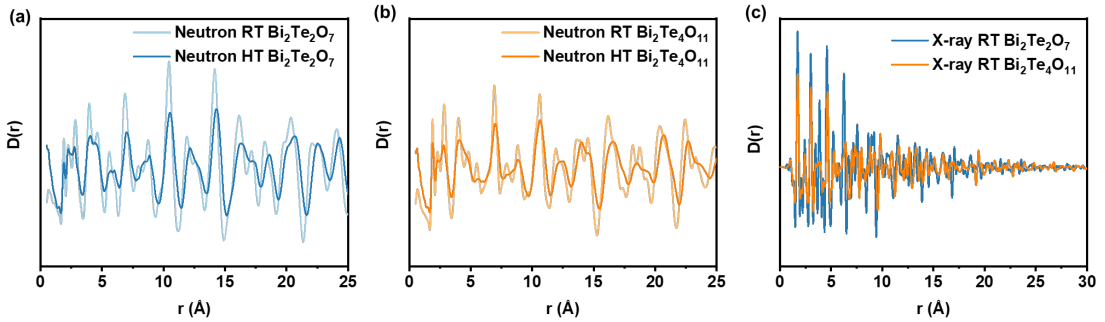

Supplementary Figure 17. Pair distribution functions of $Bi_2Te_2O_7$ and $Bi_2Te_4O_{11}$. (a) Neutron pair distribution functions of $Bi_2Te_2O_7$ at the room temperature and the high temperature 700 °C. (b) Neutron pair distribution functions of $Bi_2Te_4O_{11}$ at the room temperature and the high temperature 600 °C. (c) X-ray pair distribution functions of $Bi_2Te_2O_7$ and $Bi_2Te_4O_{11}$ at the room temperature.

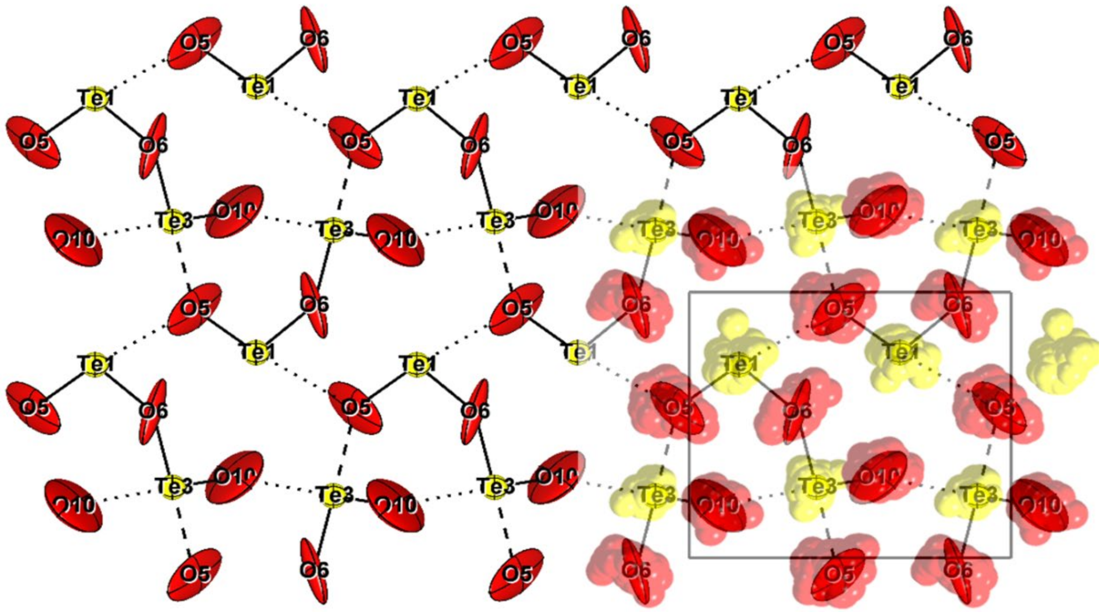

Supplementary Figure 18. Comparison between the Rietveld refined average structure model with displacement ellipsoids and the RMC refined collapsed atomic configuration in pure Te layer of $Bi_2Te_4O_{11}$ at 600 °C.

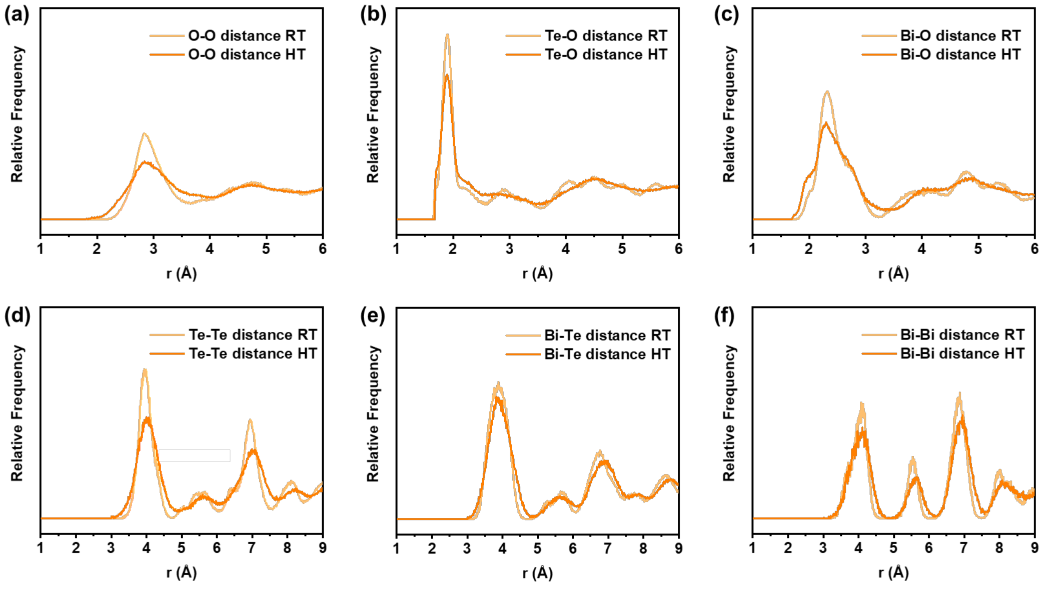

Supplementary Figure 19. Partial pair distribution functions of $Bi_2Te_4O_{11}$. Partial pair distribution functions of (a) O-O, (b) Te-O, (c) Bi-O, (d) Te-Te, (e) Bi-Te, and (f) Bi-Bi atom pairs in $Bi_2Te_4O_{11}$, representing the counted distance distribution of corresponding atom pairs from the RMC configurations.

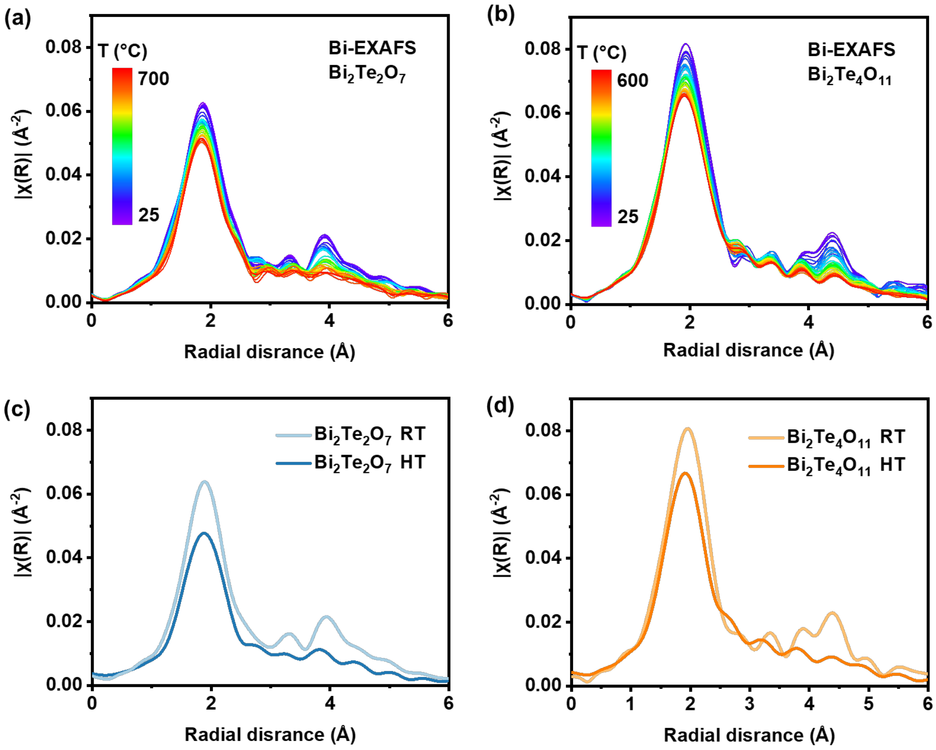

Supplementary Figure 20. Bi L_3-edge variable-temperature EXAFS spectra of $Bi_2Te_2O_7$ and $Bi_2Te_4O_{11}$. (a) Temperature evolutions of $Bi_2Te_2O_7$ from 25 °C to 650 °C. (b) Temperature evolutions of $Bi_2Te_4O_{11}$ from 25 °C to 600 °C. (c) Slow scan in $Bi_2Te_2O_7$ at 25 °C and 650 °C (d) Slow scan in $Bi_2Te_4O_{11}$ at 25 °C and 600 °C.

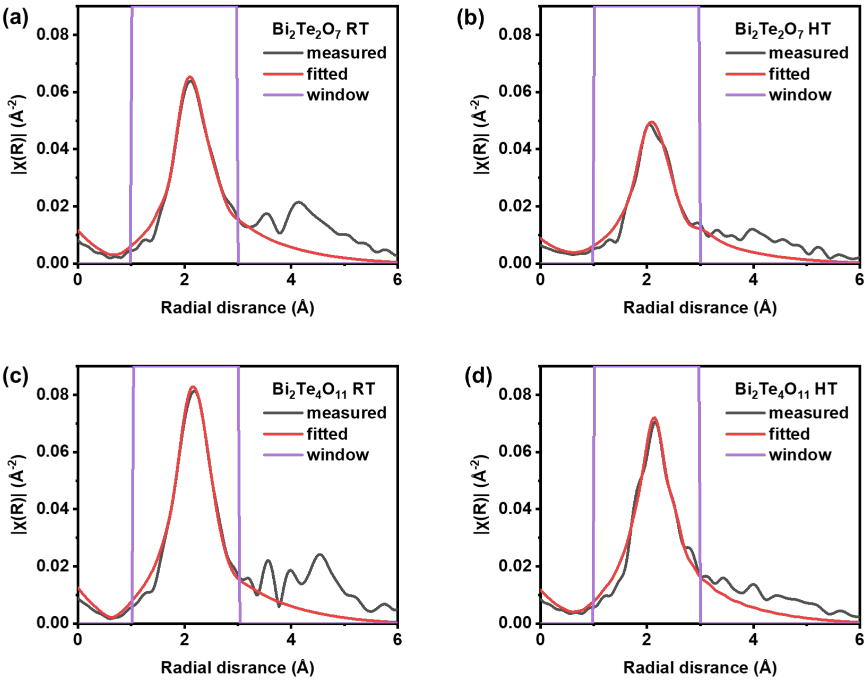

Supplementary Figure 21. The measured and fitted curves of EXAFS spectra in k^1-weighted Fourier transform R space of Bi L_3-edge for $Bi_2Te_2O_7$ and $Bi_2Te_4O_{11}$ at RT and HT.

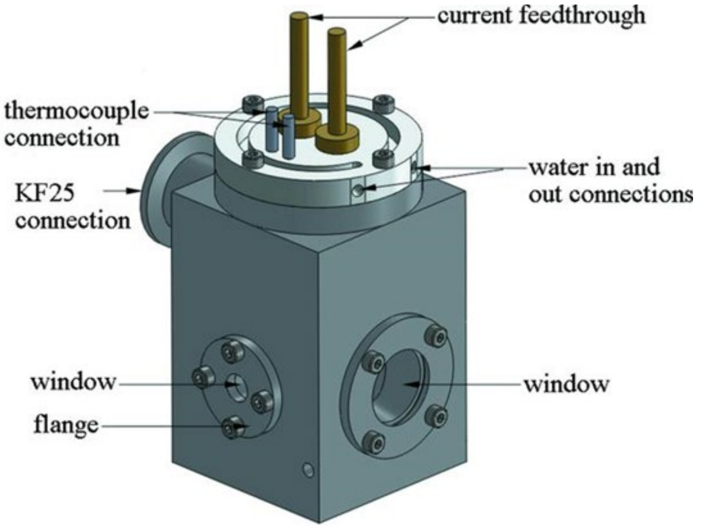

Supplementary Figure 22. Schematic diagram of heating cell for in sit X-ray absorption spectroscopy investigations[48].

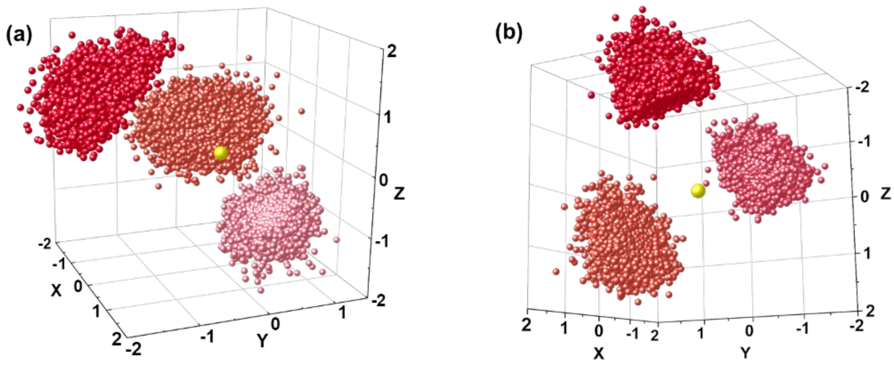

Supplementary Figure 23. Te-O bonds vector distribution in $Bi_2Te_4O_{11}$. (a) Te3-O bond vector distribution and (b) Te2-O bond vector distribution with modulus in $Bi_2Te_4O_{11}$.

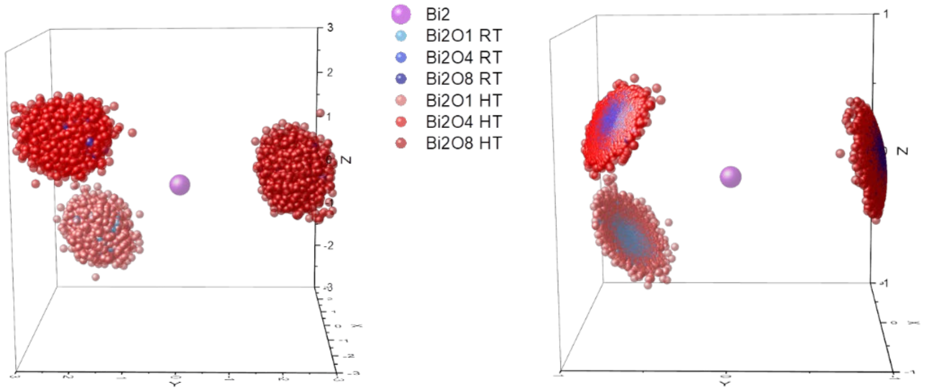

Supplementary Figure 24. Bi-O bonds vector distribution in $Bi_2Te_4O_{11}$. (a) Bi2-O bond vector distribution with modulus and (d) Bi2-O bond vector distribution in $Bi_2Te_4O_{11}$.

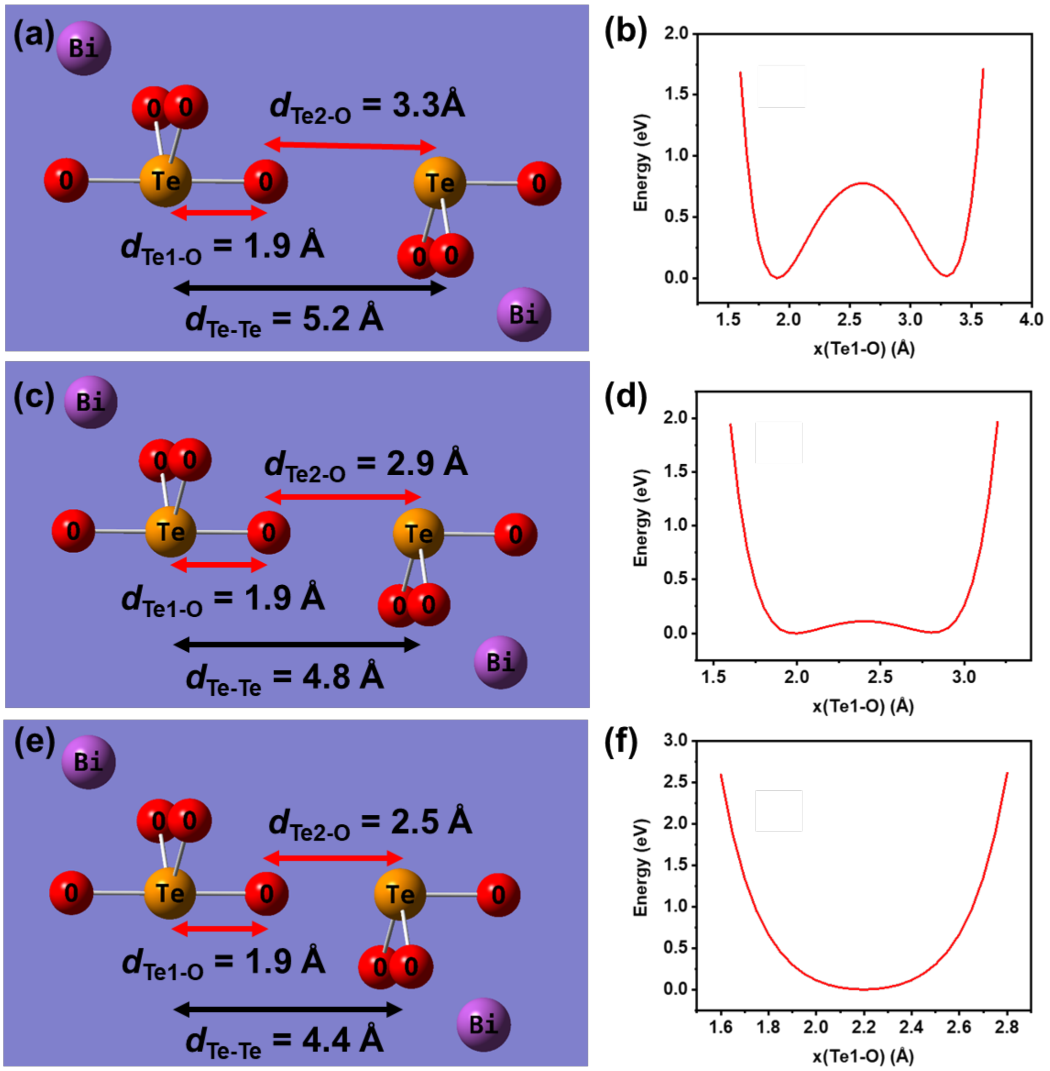

Supplementary Figure 25. Structural models (TeO_3-O-TeO_3, 2Bi) for DFT calculations and energy curves for Te1-O and Te2-O distance. The Te1-Te2 distance fixed as (a, b) 5.2 Å, (c, d) 4.8 Å and (e, f) 4.4 Å. The atom diagram is drawn with GaussView Version 6[49].

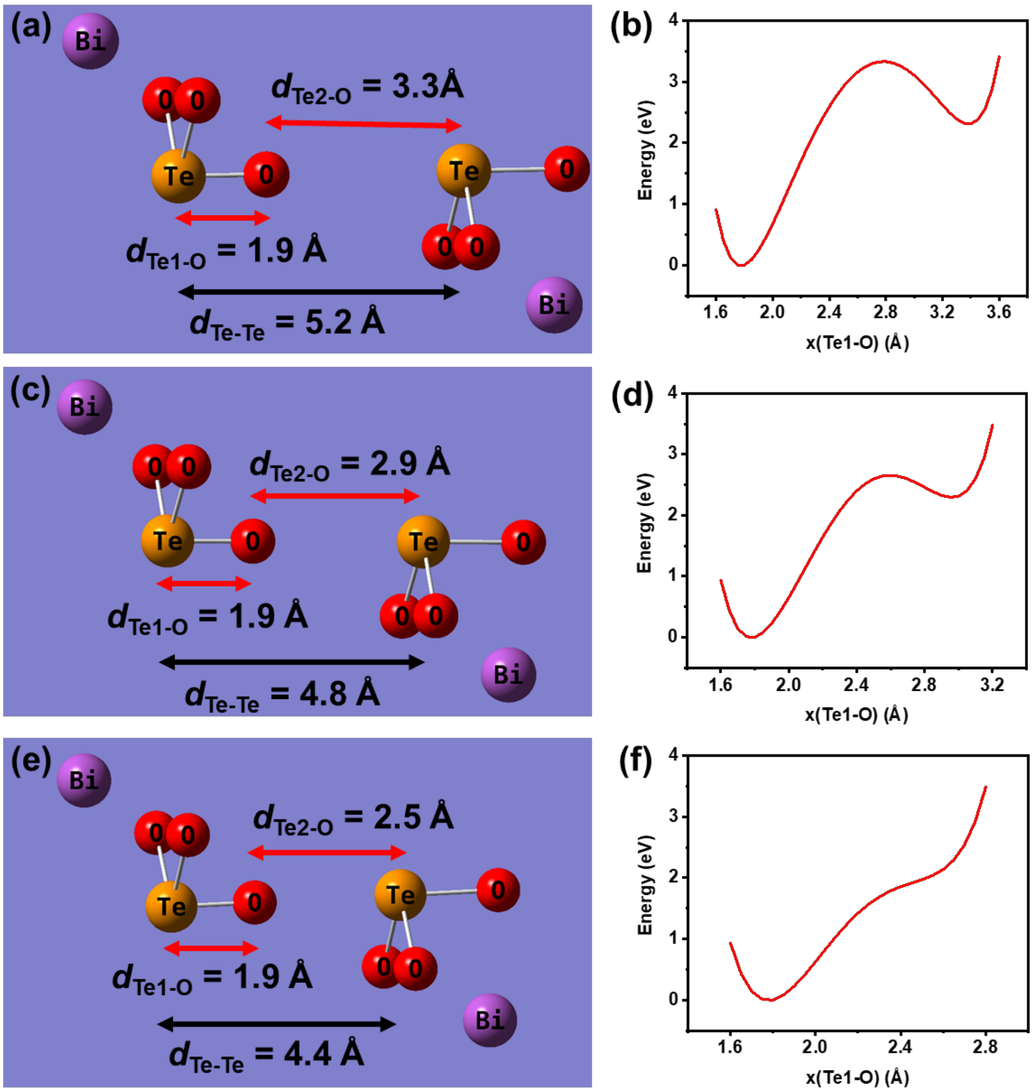

Supplementary Figure 26. Structural models (TeO3-TeO3, 2Bi) for DFT calculations and energy curves for Te1-O and Te2-O distance. The Te1-Te2 distance fixed as (a, b) 5.2 Å, (c, d) 4.8 Å and (e, f) 4.4 Å. The atom diagram is drawn with GaussView Version 6[49].

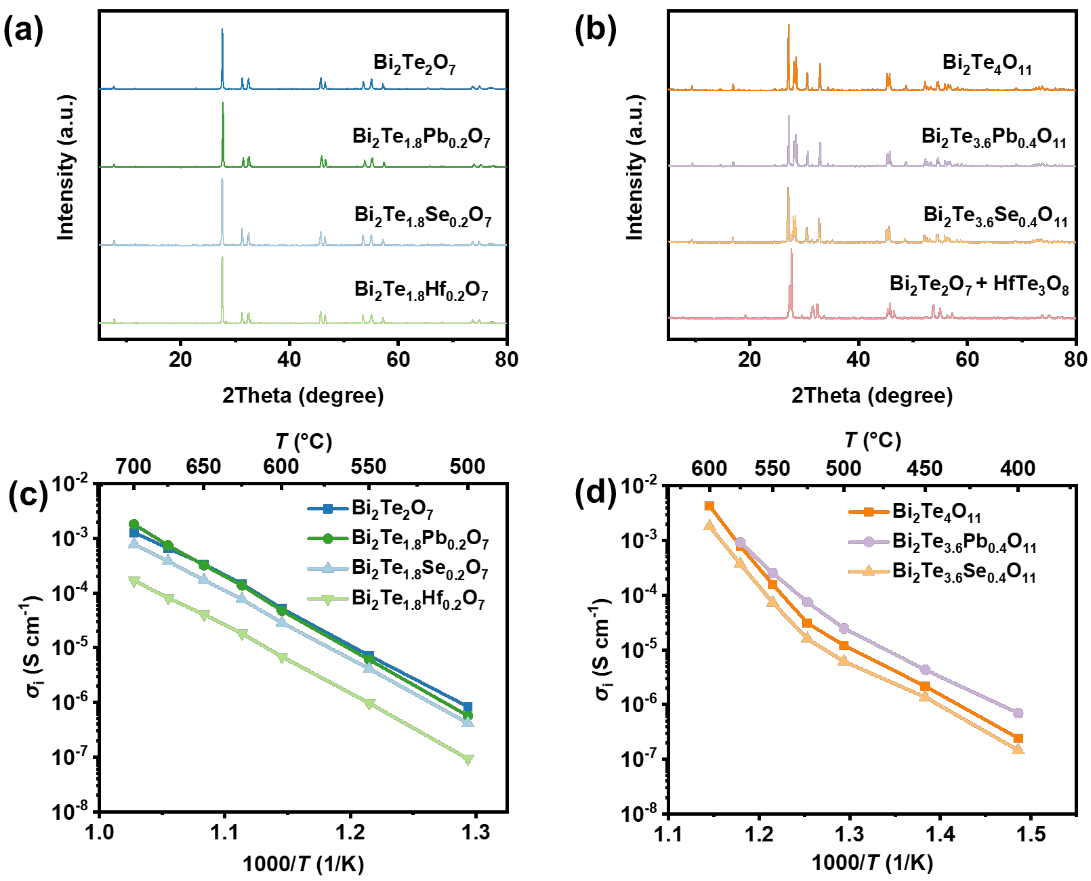

Supplementary Figure 27. Synthesis and oxygen ion conductivity of $Bi_2Te_{1.8}M_{0.2}O_7$ and $Bi_2Te_{3.6}M_{0.4}O_{11}$ (M = Pb, Se, Hf). XRD patterns of (a) $Bi_2Te_{1.8}M_{0.2}O_7$ and (b) $Bi_2Te_{3.6}M_{0.4}O_{11}$; The oxygen ion conductivity Arrhenius plots of (c) $Bi_2Te_{1.8}M_{0.2}O_7$ and (d) $Bi_2Te_{3.6}M_{0.4}O_{11}$

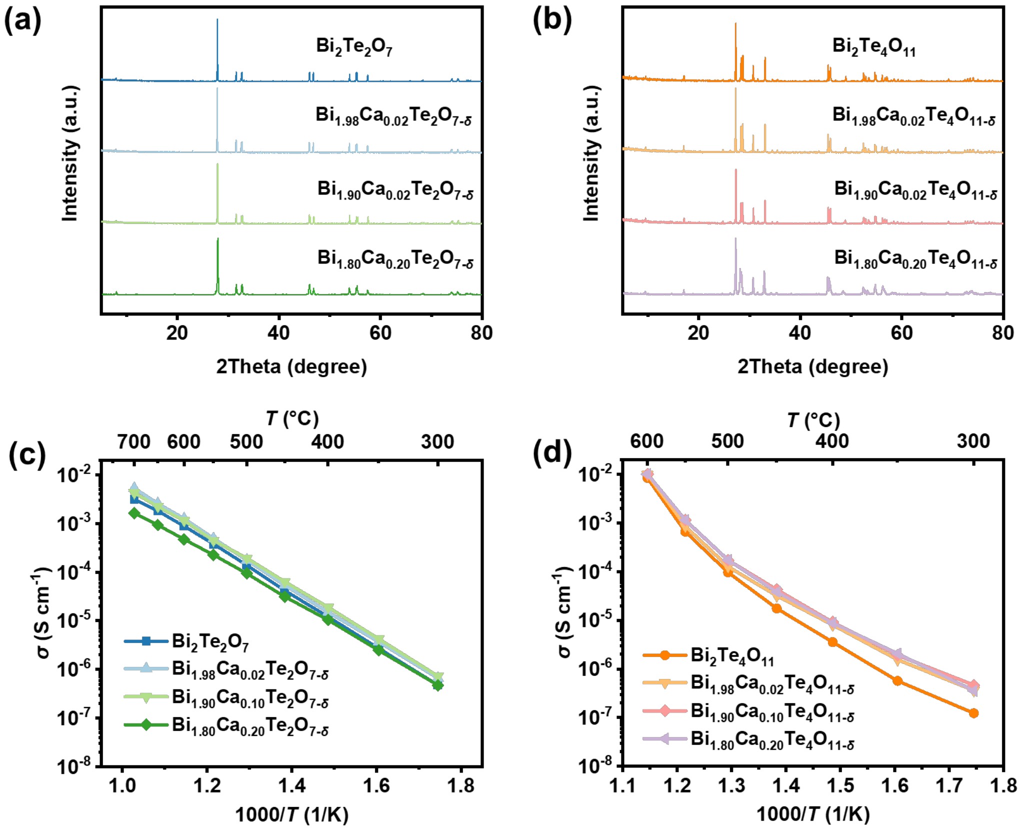

Supplementary Figure 28. Synthesis and oxygen ion conductivity of $Bi_{2-x}Ca_xTe_2O_{7-\delta}$ and $Bi_{2-x}Ca_xTe_4O_{11-\delta}$ (x = 0, 0.02, 0.10, 0.20). XRD patterns of (a) $Bi_{2-x}Ca_xTe_2O_{7-\delta}$ and (b) $Bi_{2-x}Ca_xTe_4O_{11-\delta}$; The conductivity Arrhenius plots of (c) $Bi_{2-x}Ca_xTe_2O_{7-\delta}$ and (d) $Bi_{2-x}Ca_xTe_4O_{11-\delta}$

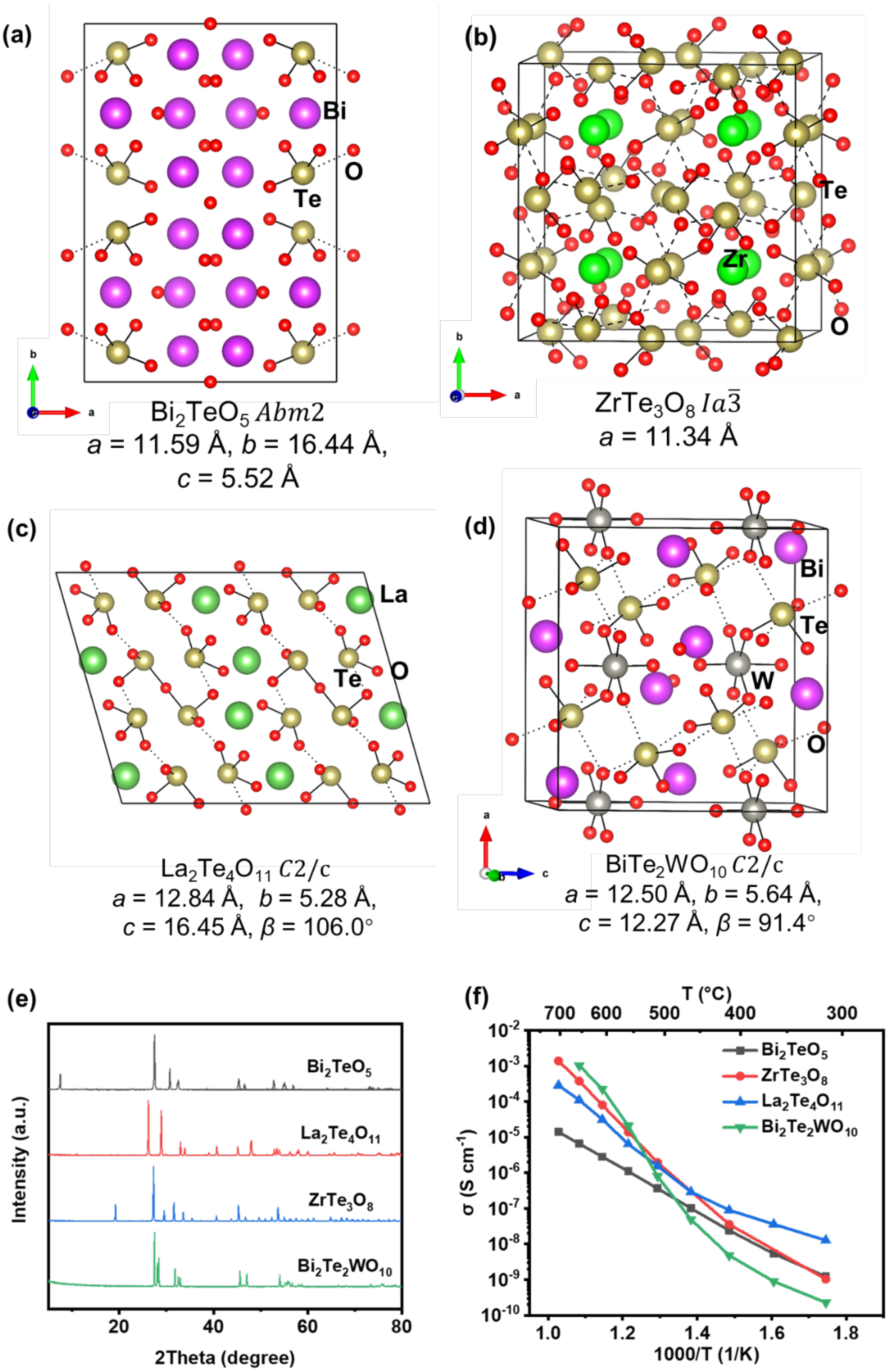

Supplementary Figure 29. Crystal structure and oxygen ion conduction of other four tellurites. (a, b, c, d) crystal structure of Bi_2TeO_5, $ZrTe_3O_8$, $La_2Te_4O_{11}$ and $Bi_2Te_2WO_{10}$. (e) XRD patterns of Bi_2TeO_5, $ZrTe_3O_8$, $La_2Te_4O_{11}$ and $Bi_2Te_2WO_{10}$. (f) Arrhenius plots of conductivity of Bi_2TeO_5, $ZrTe_3O_8$, $La_2Te_4O_{11}$ and $Bi_2Te_2WO_{10}$.

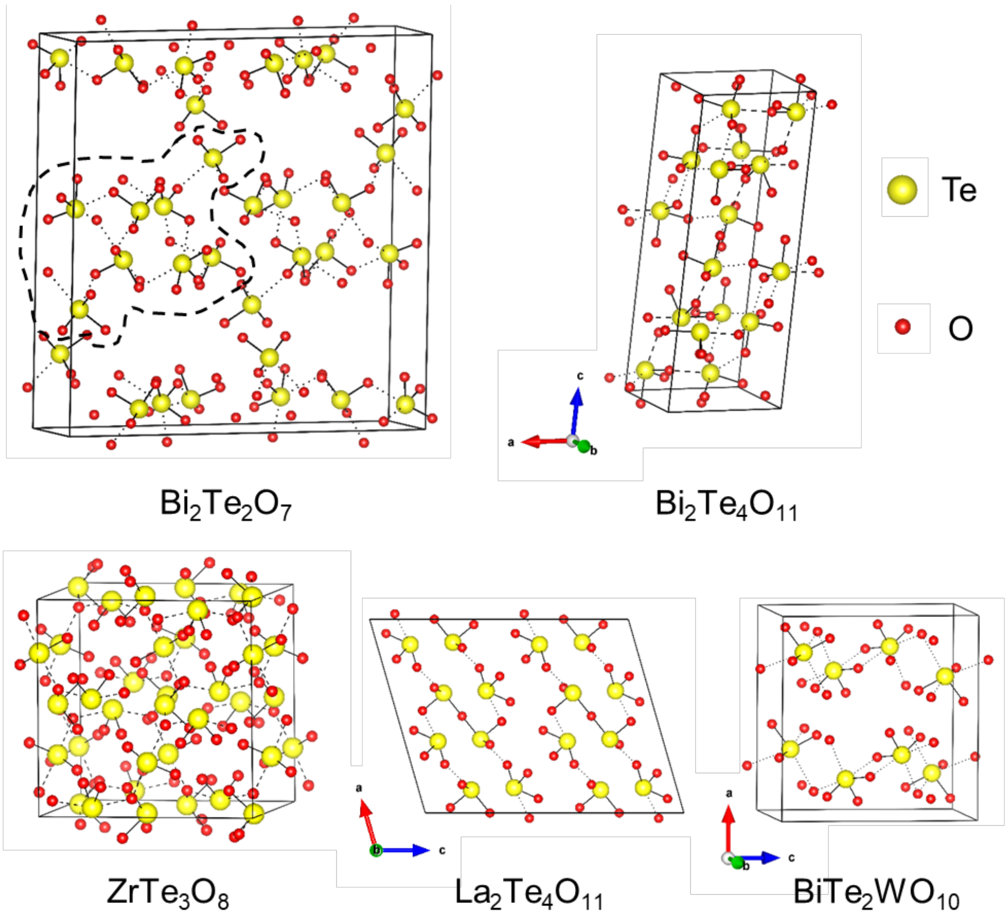

Supplementary Figure 30. Structural illustration of Te-O secondary-bond chains ($Bi_2Te_2O_7$), layers ($La_2Te_4O_{11}$) and frameworks ($Bi_2Te_4O_{11}$, $ZrTe_3O_8$, $Bi_2Te_2WO_{10}$).

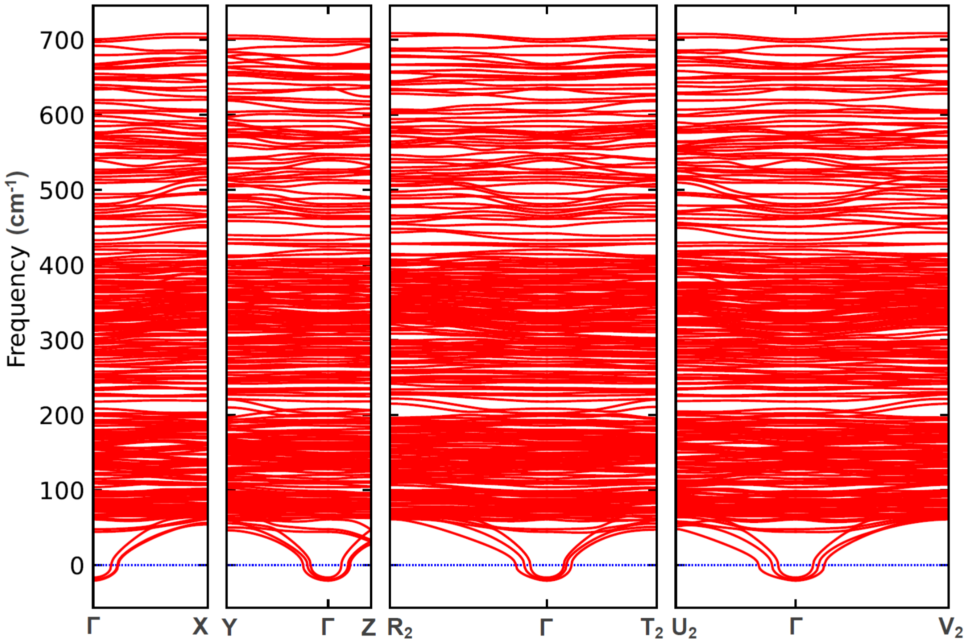

Supplementary Figure 31. Calculated phonon dispersion curve of $Bi_2Te_4O_{11}$.

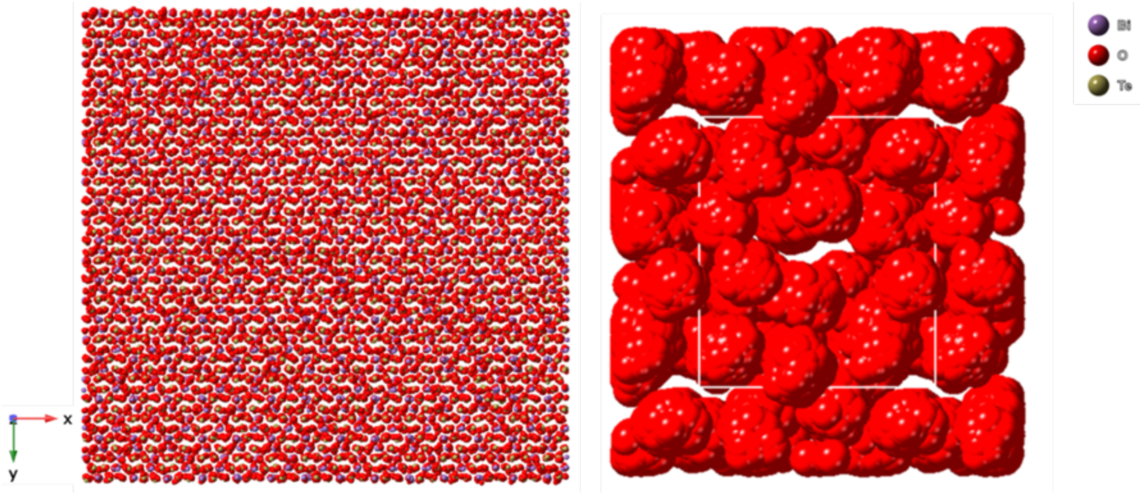

Supplementary Figure 32. **A representative MD configuration and collapse MD configuration showing the O migration path for high temperature $Bi_2Te_4O_{11}$.**

Supplementary Tables

Supplementary Table 1. Density of $Bi_2Te_2O_7$ and $Bi_2Te_4O_{11}$

Sample	Density (g cm^{-3})	Theoretical density (g cm^{-3})	Relative density
$Bi_2Te_2O_7$	7.09	7.51	94.4%
$Bi_2Te_4O_{11}$	6.54	7.01	93.3%

Supplementary Table 2. Binding energy shifts fitted from XPS spectra of Te 3d, O 1s and Bi 4f in $Bi_2Te_2O_7$, $Bi_2Te_4O_{11}$ and other Te compounds

Sample	CN(Te)	Te 3d$_{3/2}$ (eV)	Te 3d$_{5/2}$ (eV)	O 1s (eV)			Bi 4f$_{5/2}$ (eV)	Bi 4f$_{7/2}$ (eV)
TeO_2	4	586.7	576.3	531.9	530.5		-	-
$ZrTe_3O_8$	4	586.8	576.4	532.1	530.6		-	-
$Bi_2Te_2O_7$	3+1	586.4	575.9	533.0	531.5	530.1	164.4	159.1
$Bi_2Te_4O_{11}$	3+1	586.4	576.0	532.9	531.4	530.1	164.5	159.2
Na_2TeO_3	3	586.0	575.6	531.8	530.8	529.6	-	-

Supplementary Table 3. Binding energy shifts fitted from XPS spectra of Te O 1s in $Bi_2Te_2O_7$ and $Bi_2Te_4O_{11}$.

Sample	$Bi_2Te_2O_7$		$Bi_2Te_4O_{11}$	
Peak	Position (cm-1)	Area ratio (%)	Position (cm-1)	Area ratio (%)
Peak 1 (lattice oxygen)	533.0	79.7	532.9	81.3
Peak 2 (oxygen vacancies)	531.5	13.7	531.4	10.9
Peak 3 (surface-adsorbed oxygen species)	530.1	6.6	530.1	7.7

Supplementary Table 4. The symmetry splitting of $[TeO_3]$ groups.

**Polyhedral**	**Symmetry**	**Vibrational Mode**		
TeO_3^1	C_{3v}	$\nu_1\ (\nu^s_{TeO_3^1})$	$\nu_2+\nu_3\ (\nu^d_{TeO_3^1})$	
$TeO_2^1O^2$	Cs	$\nu_1\ (\nu^s_{TeO^1})$	$\nu_2\ (\nu^{as}_{TeO^1})$	$\nu_3\ (\nu_{TeO^2})$
$TeO^1O^2O^3$	C_1	$\nu_1\ (\nu_{TeO^1})$	$\nu_2\ (\nu_{TeO^2})$	$\nu_3\ (\nu_{TeO^3})$

* C_{3v} point symmetry of $[TeO_3]$ groups has irreducible representations (irreps) for the vibrational degrees of freedom $\Gamma_{vib} = 2A_1 \oplus 2E$. Both of the irreps A_1 represent a symmetric stretching vibration ν_s and a symmetric bending vibration δ_s. Both of the irreps E depict degenerate state ν_d and δ_d for the asymmetric stretching vibration ν_{as} and the asymmetric deformation vibration δ_{as}[21,50]. The corresponding four vibrations of $[TeO_3]$ ($\nu_S \approx 758$ cm^{-1}, $\nu_{as} \approx 703$ cm^{-1}, $\delta_S \approx 364$ cm^{-1} and $\delta_{as} \approx 326$ cm^{-1}) are active[50] in both the IR and Raman spectra.

Supplementary Table 5. Refined crystallographic parameters and bond valence sums (BVS) in the rietveld analysis of the combined NPD and XRD data of $Bi_2Te_2O_7$ measured at RT.

Atom	Site	*x*	*y*	*z*	Occupancy	U_{eq} & U_{iso} (Å^2)	BVS
Bi1	8*d*	0.5562(4)	0.9670(15)	0.9305(5)	1	0.0154(3)	2.95
Bi2	8*d*	0.6920(3)	0.0127(19)	0.8055(4)	1	0.0171(4)	2.81
Bi3	8*d*	0.6858(4)	-0.0155(12)	0.5557(4)	1	0.0088(6)	2.90
Bi4	8*d*	0.8057(4)	0.0193(12)	0.6803(4)	1	0.0072(4)	2.98
Te1	8*d*	0.4355(5)	0.4986(18)	0.9429(6)	1	0.0048(3)	3.90
Te2	8*d*	0.5681(5)	0.020(2)	0.6893(6)	1	0.0062(2)	3.88
Te3	8*d*	0.6883(4)	-0.4302(17)	0.4344(5)	1	0.0050(5)	3.65
Te4	8*d*	0.5668(4)	0.506(2)	0.8051(6)	1	0.0063(4)	3.91
O1	8*d*	0.7484(6)	0.2071(16)	0.7440(5)	1	0.0181(4)	-2.05
O2	8*d*	0.7371(4)	-0.284(2)	0.4954(6)	1	0.0080(6)	-1.99
O3	8*d*	0.4014(4)	0.298(2)	0.0040(5)	1	0.0270(7)	-1.91
O4	8*d*	0.4662(4)	0.752(2)	0.9929(5)	1	0.0360(3)	-1.85
O5	8*d*	0.7383(4)	-0.279(2)	0.3751(5)	1	0.0116(4)	-1.92
O6	8*d*	0.3624(4)	0.6393(16)	0.9248(6)	1	0.0162(7)	-2.06
O7	8*d*	0.6439(4)	-0.1300(17)	0.4336(5)	1	0.0102(3)	-1.94
O8	8*d*	0.6057(5)	0.6949(14)	0.8634(5)	1	0.0597(7)	-1.87
O9	8*d*	0.7386(4)	-0.186(2)	0.6240(6)	1	0.0251(5)	-2.02
O10	8*d*	0.6136(5)	0.1654(15)	0.6291(5)	1	0.0402(5)	-1.86
O11	8*d*	0.6331(4)	0.3395(19)	0.7771(5)	1	0.0155(8)	-2.00

Atom	U_{11} (Å^2)	U_{22} (Å^2)	U_{33} (Å^2)	U_{12} (Å^2)	U_{13} (Å^2)	U_{23} (Å^2)
O1	0.0140(4)	0.0170(4)	0.0233(2)	-0.0018(6)	0.0139(5)	-0.0082(3)
O2	0.01595(18)	0.0066(9)	0.0014(8)	-0.0007(2)	0.00065(13)	0.0014(4)
O3	0.01464(12)	0.0525(9)	0.0140(7)	-0.0210(9)	0.0096(2)	-0.0200(2)
O4	0.0470(3)	0.0098(5)	0.0512(4)	-0.00424(12)	-0.0366(5)	-0.0011(2)
O5	0.0056(2)	0.0271(3)	0.0020(8)	0.0032(4)	0.0020(5)	-0.0024(6)
O6	0.0063(6)	0.0120(4)	0.0305(15)	-0.0018(5)	-0.0006(9)	0.00586(13)
O7	0.00501(10)	0.01704(11)	0.0085(9)	-0.0006(2)	-0.00044(15)	0.0045(8)
O8	0.0943(9)	0.0499(7)	0.0348(7)	-0.0333(8)	0.0220(8)	-0.0084(3)
O9	0.0088(4)	0.0477(5)	0.0190(4)	-0.00080(11)	0.0050(8)	0.0141(5)
O10	0.0152(4)	0.0304(2)	0.0751(8)	-0.0074(2)	0.0081(9)	-0.0108(6)
O11	0.0264(4)	0.0120(8)	0.0079(9)	-0.0044(6)	0.0075(3)	0.0041(5)
O12	0.0241(4)	0.0126(2)	0.0391(3)	0.0031(3)	-0.0270(7)	-0.0081(3)
O13	0.0301(5)	0.0180(5)	0.0323(8)	0.0025(2)	0.0123(4)	0.0071(5)
O14	0.0341(8)	0.0191(6)	0.0052(5)	0.0051(8)	-0.0033(4)	-0.0035(5)

* Space group: *Pbcn*; Number of formulas per unit cell: Z = 8; Lattice parameters: *a* = 22.7884(6) Å, *b* = 5.51467(18) Å, *c* = 22.1279(7) Å; Profile *R* factor: R_p = 4.59%, Weighted profile *R* factor: R_{wp} = 7.02% and Goodness of fit: GOF = 2.14.

** U_{iso} denotes the isotropic atomic displacement parameter. U_{eq} stands for Equivalent isotropic atomic displacement parameter (ADP). BVS means bond valence sums.

Supplementary Table 6. Refined crystallographic parameters and bond valence sums (BVS) in the rietveld analysis of the combined NPD and XRD data of $Bi_2Te_2O_7$ measured at 700 °C.

Atom	Site	*x*	*y*	*z*	Occupancy	U_{eq} & U_{iso} (Å^2)	BVS
Bi1	8*d*	0.5596(5)	0.968(2)	0.9425(7)	1	0.0191(5)	3.11
Bi2	8*d*	0.6954(6)	0.012(2)	0.8074(4)	1	0.0283(8)	2.81
Bi3	8*d*	0.6902(6)	-0.0126(17)	0.5566(5)	1	0.0108(6)	2.73
Bi4	8*d*	0.8058(7)	0.015(2)	0.6831(6)	1	0.0072(4)	2.81
Te1	8*d*	0.4362(9)	0.499(3)	0.9418(9)	1	0.0090(5)	3.51
Te2	8*d*	0.5675(10)	0.021(3)	0.6890(9)	1	0.0064(3)	3.80
Te3	8*d*	0.6894(8)	-0.430(2)	0.4380(8)	1	0.0111(3)	3.22
Te4	8*d*	0.5674(10)	0.503(3)	0.8071(7)	1	0.0049(7)	3.76
O1	8*d*	0.7491(8)	0.206(3)	0.7456(8)	1	0.0159(6)	-2.05
O2	8*d*	0.7411(5)	-0.282(3)	0.4976(8)	1	0.0098(3)	-1.98
O3	8*d*	0.4033(5)	0.296(3)	0.0074(5)	1	0.0498(4)	-2.06
O4	8*d*	0.4637(5)	0.752(2)	0.9904(5)	1	0.0694(2)	-2.05
O5	8*d*	0.7363(5)	-0.267(3)	0.3729(7)	1	0.0542(8)	-1.81
O6	8*d*	0.3593(6)	0.593(2)	0.9148(6)	1	0.0255(4)	-1.68
O7	8*d*	0.6462(5)	-0.1303(17)	0.4346(8)	1	0.0251(5)	-1.89
O8	8*d*	0.6070(6)	0.696(2)	0.8622(5)	1	0.0444(5)	-1.68
O9	8*d*	0.7401(6)	-0.190(2)	0.6202(7)	1	0.0191(4)	-1.94
O10	8*d*	0.6135(5)	0.164(2)	0.6304(6)	1	0.0587(6)	-1.77
O11	8*d*	0.6346(5)	0.342(2)	0.7751(7)	1	0.0280(3)	-1.79

Atom	U_{11} (Å^2)	U_{22} (Å^2)	U_{33} (Å^2)	U_{12} (Å^2)	U_{13} (Å^2)	U_{23} (Å^2)
O1	0.0030(8)	0.0117(5)	0.0331(6)	0.00302(11)	0.0050(5)	0.0027(4)
O2	0.0169(6)	0.0076(2)	0.0049(9)	-0.0010(2)	0.00081(9)	0.00164(19)
O3	0.0911(9)	0.0484(8)	0.0098(2)	0.0062(3)	-0.0166(2)	-0.0045(5)
O4	0.0932(6)	0.0905(2)	0.0246(5)	-0.0229(7)	-0.0119(9)	-0.0118(8)
O5	0.0694(11)	0.0630(9)	0.0302(7)	0.0228(3)	0.0129(2)	0.0116(8)
O6	0.0241(7)	0.0222(3)	0.0303(3)	0.0041(2)	0.0126(19)	0.0140(3)
O7	0.0212(3)	0.0058(9)	0.0482(8)	0.0080(5)	-0.0028(2)	0.0041(3)
O8	0.0560(7)	0.0717(8)	0.0055(3)	-0.0158(6)	0.0093(9)	-0.0099(7)
O9	0.0243(7)	0.0020(2)	0.0310(7)	-0.00356(14)	0.0137(6)	-0.0029(5)
O10	0.0405(4)	0.0369(9)	0.0988(14)	-0.0193(6)	0.0316(5)	-0.0302(3)
O11	0.0126(5)	0.0496(3)	0.0217(5)	0.0125(2)	0.0082(8)	0.0163(6)
O12	0.0178(8)	0.0587(8)	0.0888(15)	0.0180(7)	-0.0150(8)	0.0099(6)
O13	0.0413(7)	0.0218(9)	0.0406(12)	0.0087(2)	0.0205(6)	0.0149(10)
O14	0.0344(4)	0.0390(5)	0.0052(3)	0.0091(7)	-0.0033(6)	-0.0035(7)

* Space group: *Pbcn*; Number of formulas per unit cell: Z = 8; Lattice parameters: *a* = 22.9469(10) Å, *b* = 5.5890(2) Å, *c* = 22.4510(9) Å; Profile *R* factor: R_p = 5.54%, Weighted profile *R* factor: R_{wp} = 7.69% and Goodness of fit: GOF = 2.54.

Supplementary Table 7. Refined crystallographic parameters and bond valence sums (BVS) in the rietveld analysis of the combined NPD and XRD data of $Bi_2Te_4O_{11}$ measured at RT.

Atom	Site	*x*	*y*	*z*	Occupancy	U_{eq} & U_{iso} (Å^2)	BVS
Bi1	4*e*	0.5631(2)	0.1188(3)	0.42323(10)	1	0.0107(4)	3.14
Bi2	4*e*	0.5658(2)	0.1128(2)	0.91339(9)	1	0.0086(3)	2.95
Te1	4*e*	0.2787(4)	0.1562(4)	0.24770(15)	1	0.0055(3)	3.94
Te2	4*e*	0.9121(4)	0.1184(3)	0.57415(13)	1	0.0056(4)	3.87
Te3	4*e*	0.2404(3)	0.0846(3)	0.75571(13)	1	0.0047(2)	3.81
Te4	4*e*	0.8980(3)	0.1414(3)	0.07265(11)	1	0.0051(2)	3.93
O1	4*e*	0.9403(4)	0.1733(3)	0.47792(13)	1	0.0119(7)	-2.01
O2	4*e*	0.9185(4)	0.1745(3)	0.97019(15)	1	0.0185(3)	-2.04
O3	4*e*	0.2640(4)	0.1567(3)	0.58430(12)	1	0.0158(3)	-1.86
O4	4*e*	0.2717(4)	0.1433(4)	0.34504(13)	1	0.0188(6)	-1.98
O5	4*e*	0.5606(4)	0.0269(4)	0.76337(14)	1	0.0116(5)	-1.92
O6	4*e*	0.9540(4)	0.1533(3)	0.75196(13)	1	0.0176(6)	-1.91
O7	4*e*	0.6369(5)	0.0662(3)	0.54179(16)	1	0.0136(7)	-2.25
O8	4*e*	0.3531(4)	0.1685(3)	0.10722(13)	1	0.0208(6)	-1.92
O9	4*e*	0.6731(4)	0.0198(3)	0.04019(15)	1	0.0114(5)	-2.14
O10	4*e*	0.8001(4)	0.1016(3)	0.18796(12)	1	0.0217(10)	-1.80
O11	4*e*	0.3145(4)	0.2256(3)	0.83462(14)	1	0.0143(5)	-1.81

Atom	U_{11} (Å^2)	U_{22} (Å^2)	U_{33} (Å^2)	U_{12} (Å^2)	U_{13} (Å^2)	U_{23} (Å^2)
O1	0.01171(11)	0.0176(4)	0.0064(3)	-0.0042(4)	-0.00173(16)	0.00080(19)
O2	0.0132(5)	0.0289(4)	0.0135(2)	-0.00950(11)	0.00633(10)	0.0062(7)
O3	0.0300(6)	0.0077(3)	0.0099(2)	-0.0051(8)	0.0033(4)	-0.0045(8)
O4	0.0179(9)	0.0210(5)	0.0176(6)	-0.0055(7)	-0.0066(4)	-0.0083(6)
O5	0.0099(7)	0.0088(8)	0.0162(3)	0.0066(4)	-0.0036(8)	0.00034(8)
O6	0.0086(5)	0.0278(8)	0.0164(5)	0.0072(5)	0.0016(2)	-0.0036(7)
O7	0.0104(7)	0.0203(2)	0.0103(3)	-0.0040(4)	-0.0021(4)	-0.0072(4)
O8	0.0211(5)	0.0105(7)	0.0310(2)	0.0092(6)	-0.0084(4)	0.0009(2)
O9	0.0121(6)	0.0177(8)	0.0042(6)	-0.00852(17)	-0.0021(4)	0.0073(6)
O10	0.0125(11)	0.0138(9)	0.0389(13)	0.0018(4)	-0.00618(15)	0.0201(3)
O11	0.0169(8)	0.0202(9)	0.0059(8)	-0.00021(9)	-0.0041(4)	0.0022(2)

* Space group: $P2_1/n$; Number of formulas per unit cell: Z = 4; Lattice parameters: a = 6.9946(2) Å, b = 7.96406(17) Å, c = 18.8624(9) Å, β = 95.280(3) °; Profile R factor: R_p = 3.16%, Weighted profile R factor: R_{wp} = 5.01% and Goodness of fit: GOF = 2.21.

Supplementary Table 8. Refined crystallographic parameters and bond valence sums (BVS) in the Rietveld analysis of the combined NPD and XRD data of $Bi_2Te_4O_{11}$ measured at 600 °C.

Atom	Site	x	y	z	Occupancy	U_{eq} & U_{iso} (Å^2)	BVS
Bi1	4*e*	0.5740(5)	0.1190(6)	0.4224(2)	1	0.0091(4)	2.91
Bi2	4*e*	0.5622(6)	0.1112(6)	0.9166(2)	1	0.0107(4)	2.76
Te1	4*e*	0.2726(8)	0.1548(9)	0.2481(3)	1	0.0051(5)	3.55
Te2	4*e*	0.9035(8)	0.1190(7)	0.5736(3)	1	0.0045(5)	3.62
Te3	4*e*	0.2338(8)	0.1044(7)	0.7569(3)	1	0.0101(4)	3.56
Te4	4*e*	0.9017(7)	0.1468(7)	0.0710(3)	1	0.0057(3)	3.67
O1	4*e*	0.9416(9)	0.1751(7)	0.4741(3)	1	0.0315(5)	-1.86
O2	4*e*	0.9343(8)	0.1769(8)	0.9752(3)	1	0.0269(5)	-1.89
O3	4*e*	0.2754(7)	0.1595(7)	0.5922(3)	1	0.0146(6)	-1.76
O4	4*e*	0.2709(8)	0.1180(8)	0.3447(3)	1	0.0218(8)	-1.82
O5	4*e*	0.5636(8)	0.0364(8)	0.7671(3)	1	0.064(3)	-1.67
O6	4*e*	0.9505(11)	0.1652(11)	0.7523(4)	1	0.0304(6)	-1.81
O7	4*e*	0.6354(9)	0.0586(7)	0.5363(3)	1	0.0207(7)	-2.08
O8	4*e*	0.3246(8)	0.1648(7)	0.1061(3)	1	0.0262(7)	-1.82
O9	4*e*	0.6556(9)	0.0359(8)	0.0415(3)	1	0.0414(10)	-2.00
O10	4*e*	0.8035(10)	0.0880(10)	0.1939(3)	1	0.089(5)	-1.69
O11	4*e*	0.2994(8)	0.2262(6)	0.8384(3)	1	0.0283(7)	-1.67

Atom	U_{11} (Å^2)	U_{22} (Å^2)	U_{33} (Å^2)	U_{12} (Å^2)	U_{13} (Å^2)	U_{23} (Å^2)
O1	0.0335(8)	0.0454(6)	0.0157(9)	-0.0195(8)	-0.0133(9)	0.0113(6)
O2	0.0285(4)	0.0398(5)	0.0125(8)	-0.0168(6)	0.0042(9)	0.0050(7)
O3	0.0217(9)	0.0071(5)	0.0150(5)	-0.0047(5)	0.0026(2)	-0.0023(3)
O4	0.0202(9)	0.0239(6)	0.0213(14)	-0.0077(7)	-0.0102(10)	-0.0078(7)
O5	0.0410(11)	0.0404(15)	0.110(4)	0.0245(13)	-0.0370(8)	-0.0632(4)
O6	0.066(3)	0.0096(5)	0.0153(5)	0.0175(9)	-0.0094(6)	-0.0049(3)
O7	0.0156(7)	0.0313(6)	0.0152(9)	-0.0061(2)	-0.0100(4)	-0.0078(5)
O8	0.0226(12)	0.0156(10)	0.0405(6)	0.0088(5)	-0.0161(9)	0.0098(7)
O9	0.0344(19)	0.0546(6)	0.0353(13)	-0.0108(5)	0.0087(3)	0.0109(9)
O10	0.045(3)	0.0469(17)	0.175(7)	0.0229(9)	-0.0281(16)	0.0453(3)
O11	0.0404(6)	0.0287(5)	0.0158(8)	-0.0170(3)	0.0126(6)	-0.0106(7)

* Space group: $P2_1/n$; Number of formulas per unit cell: Z = 4; Lattice parameters: a = 7.0599(3) Å, b = 8.0444(4) Å, c = 19.1744(15) Å, β = 94.731(5) °; Profile R factor: R_p = 3.97%, Weighted profile R factor: R_{wp} = 6.44% and Goodness of fit: GOF = 2.41.

Supplementary Table 9. Inter-atomic distances, angles and solid angle of Te-O and Bi-O bonds in $Bi_2Te_2O_7$ for the average structural model at 25℃

Te1	Distance (Å)	Te1	Angle (°)	Solid Angle (°)
Te1-O6	1.882(15)			19.9
Te1-O3	1.912(17)			19.5
Te1-O4	1.915(16)			22.2
Te1···O14	2.628(16)	O3-Te1···O14	173.8(8)	12.1
Te1···O10	2.678(16)	O4-Te1···O10	176.0(7)	10.4
Te1 · · · · O4	2.990(16)	O6-Te1· · · ·O13	162.8(8)	3.5
Te1· · · ·O13	3.310(17)	O6-Te1· · · ·O13	130.6(8)	3.8
Te2	Distance (Å)	Te2	Angle (°)	Solid Angle (°)
Te2-O14	1.869(16)			22.8
Te2-O10	1.869(17)			22.4
Te2-O12	1.879(16)			22.0
Te2···O13	2.879(17)	O12-Te2···O13	171.4(8)	11.9
Te2···O11	3.010(16)	O14-Te2···O11	169.1(8)	8.3
Te3	Distance (Å)	Te3	Angle (°)	Solid Angle (°)
Te3-O2	1.926(16)			18.8
Te3-O5	1.928(15)			19.0
Te3-O7	1.941(14)			19.6
Te3···O8	2.854(15)	O2-Te3···O8	168.8(7)	11.7
Te3· · · ·O5	2.865(15)	O7-Te3· · · ·O5	150.8(7)	4.4
Te3···O3	2.879(14)	O5-Te3···O3	165.4(7)	11
Te3· · · ·O6	3.342(17)	O5-Te3· · · ·O6	142.1(6)	2.9
Te4	Distance (Å)	Te4	Angle (°)	Solid Angle (°)
Te4-O13	1.862(16)			22.9
Te4-O11	1.874(15)			21.6
Te4-O8	1.881(16)			21.3
Te4···O14	2.859(15)	O11-Te4···O14	176.0(7)	11.4
Te4···O12	2.917(16)	O13-Te4···O12	170.8(7)	9
Bi1	Distance (Å)	Bi2	Distance (Å)	
Bi1-O7	2.194(13)	Bi2-O1	2.158(15)	
Bi1-O3	2.274(15)	Bi2-O11	2.334(14)	
Bi1-O4	2.353(15)	Bi2-O5	2.376(15)	
Bi1-O8	2.394(14)	Bi2-O12	2.512(14)	
Bi1-O13	2.395(15)	Bi2-O1	2.557(15)	
Bi1-O4	2.743(14)	Bi2-O5	2.561(14)	
Bi1-O14	2.787(15)	Bi2-O8	2.930(14)	
Bi3	Distance (Å)	Bi4	Distance (Å)	
Bi3-O9	2.149(15)	Bi4-O1	2.183(15)	
Bi3-O6	2.241(12)	Bi4-O9	2.275(14)	
Bi3-O2	2.311(14)	Bi4-O9	2.284(14)	
Bi3-O10	2.518(14)	Bi4-O12	2.492(14)	
Bi3-O2	2.550(14)	Bi4-O1	2.544(14)	

Bi3-O9	2.926(14)	Bi4-O6	2.741(16)
Bi3-O7	2.934(14)	Bi4-O11	2.742(14)
Bi3-O3	2.948(14)	Bi4-O10	2.912(13)

* Te-O denotes the covalent bonds, Te···O denotes the secondary bonds and Te· · · ·O suggests failing to meet the criteria for secondary bonds.

Supplementary Table 10. Inter-atomic distances, angles and solid angle of Te-O and Bi-O bonds in $Bi_2Te_2O_7$ for the average structural model at 700°C

Te1	Distance (Å)	Te1	Angle (°)	Solid Angle (°)
Te1-O6	1.89(3)			19.9
Te1-O3	2.01(3)			19.5
Te1-O4	1.94(3)			22.2
Te1···O14	2.66(3)	O3-Te1···O14	175.1(12)	12.1
Te1···O10	2.73(3)	O4-Te1···O10	173.9(11)	10.4
Te1 · · · · O4	3.09(3)	O6-Te1· · · ·O13	162.4(11)	3.5
Te1· · · ·O13	3.30(3)	O6-Te1· · · ·O13	123.8(10)	3.8
**Te2**	**Distance (Å)**	**Te2**	**Angle (°)**	**Solid Angle (°)**
Te2-O14	1.86(3)			23.0
Te2-O10	1.87(3)			22.7
Te2-O12	1.91(3)			21.8
Te2···O13	2.90(3)	O12-Te2···O13	171.4(12)	12.0
Te2···O11	3.05(3)	O14-Te2···O11	169.0(11)	8.2
**Te3**	**Distance (Å)**	**Te3**	**Angle (°)**	**Solid Angle (°)**
Te3-O2	1.97(3)			19.0
Te3-O5	1.97(3)			17.9
Te3-O7	1.948(17)			19.9
Te3···O8	2.95(3)	O2-Te3···O8	172.1(10)	11.0
Te3· · · ·O5	2.93(3)	O7-Te3· · · ·O5	146.0(11)	4.2
Te3···O3	2.89(3)	O5-Te3···O3	158.7(10)	11.4
Te3· · · ·O6	3.49(3)	O5-Te3· · · ·O6	146.6(8)	2.5
**Te4**	**Distance (Å)**	**Te4**	**Angle (°)**	**Solid Angle (°)**
Te4-O13	1.86(3)			23.7
Te4-O11	1.92(3)			21.9
Te4-O8	1.88(3)			21.5
Te4···O14	2.89(3)	O11-Te4···O14	171.7(11)	11.5
Te4···O12	2.97(3)	O13-Te4···O12	175.7(11)	8.7
**Bi1**	**Distance (Å)**	**Bi2**	**Distance (Å)**	
Bi1-O7	2.192(17)	Bi2-O1	2.15(3)	
Bi1-O3	2.04(2)	Bi2-O11	2.424(17)	
Bi1-O4	2.237(18)	Bi2-O5	2.253(19)	
Bi1-O8	2.597(18)	Bi2-O12	2.614(18)	
Bi1-O13	2.63(3)	Bi2-O1	2.54(3)	
Bi1-O4	2.731(17)	Bi2-O5	2.548(19)	
Bi1-O14	3.06(2)	Bi2-O8	2.958(18)	

Bi3	Distance (Å)	Bi4	Distance (Å)
Bi3-O9	2.082(19)	Bi4-O1	2.19(3)
Bi3-O6	2.561(16)	Bi4-O9	2.36(2)
Bi3-O2	2.32(2)	Bi4-O9	2.413(19)
Bi3-O10	2.611(18)	Bi4-O12	2.524(19)
Bi3-O2	2.43(2)	Bi4-O1	2.56(3)
Bi3-O9	2.801(18)	Bi4-O6	2.55(2)
Bi3-O7	2.99(3)	Bi4-O11	2.66(2)
Bi3-O3	3.105(18)	Bi4-O10	2.946(18)

* Te-O denotes the covalent bonds, Te···O denotes the secondary bonds and Te· · ·O suggests failing to meet the criteria for secondary bonds.

Supplementary Table 11. Inter-atomic distances, angles and solid angle of Te-O and Bi-O bonds in $Bi_2Te_4O_{11}$ for the average structural model at 25℃

Te1	Distance (Å)	Te1	Angle (°)	Solid Angle (°)
Te1-O4	1.843(4)			21.8
Te1-O5	1.864(5)			21.2
Te1-O6	1.947(4)			20.1
Te1···O8	2.747(4)	O4-Te1···O8	170.58(18)	11.4
Te1···O5	2.977(5)	O5-Te1···O5	173.50(18)	9.1
Te1· · ·O6	2.982(4)	O6-Te1· · ·O6	174.13(16)	4.2
Te1· · ·O10	3.456(4)	O6-Te1· · ·O10	134.24(16)	3.5
**Te2**	**Distance (Å)**	**Te2**	**Angle (°)**	**Solid Angle (°)**
Te2-O8	1.867(4)			21.7
Te2-O1	1.894(4)			19.1
Te2-O7	2.007(5)			18.1
Te2···O3	2.468(4)	O7-Te2···O3	165.78(17)	14.3
Te2···O1	2.759(4)	O8-Te2···O1	170.23(18)	10.5
Te2· · ·O4	2.945(4)	O1-Te2· · ·O4	138.40(15)	8.9
Te2· · ·O6	3.351(4)	O1-Te2· · ·O6	158.81(15)	2.2
Te2· · ·O9	3.500(4)	O4-Te2· · ·O9	159.45(11)	0.1
**Te3**	**Distance (Å)**	**Te3**	**Angle (°)**	**Solid Angle (°)**
Te3-O10	1.861(4)			20.6
Te3-O11	1.897(4)			19.2
Te3-O6	2.070(4)			19.5
Te3···O5	2.276(4)	O6-Te3···O5	175.98(17)	16.3
Te3···O10	2.855(4)	O10-Te3···O10	171.59(15)	9.5
Te3· · ·O3	3.303(4)	O3-Te3· · ·O10	136.17(15)	4.4
Te3· · ·O11	3.331(4)	O11-Te3· · ·O11	156.75(14)	3.4
**Te4**	**Distance (Å)**	**Te4**	**Angle (°)**	**Solid Angle (°)**
Te4-O3	1.884(4)			19.7
Te4-O9	1.899(4)			19.0
Te4-O2	1.967(4)			18.4

Te4···O10	2.359(4)	O2-Te4···O10	167.37(16)	15.6
Te4· · · ·O7	2.954(4)	O9-Te4···O7	143.56(15)	4.9
Te4···O2	2.969(4)	O3-Te4···O2	170.65(14)	9.8
Te4· · · ·O8	3.194(4)	O9-Te4· · · ·O8	151.55(15)	4.2
Bi1	Distance (Å)	Bi2	Distance (Å)	
Bi1-O2	2.163(4)	Bi2-O9	2.222(4)	
Bi1-O7	2.175(4)	Bi2-O1	2.312(4)	
Bi1-O7	2.285(4)	Bi2-O8	2.352(3)	
Bi1-O4	2.409(3)	Bi2-O11	2.370(3)	
Bi1-O3	2.515(4)	Bi2-O9	2.548(4)	
Bi1-O1	2.775(4)	Bi2-O2	2.642(4)	
Bi1-O11	2.820(4)	Bi2-O4	2.801(4)	
		Bi2-O5	2.906(4)	

* Te-O denotes the covalent bonds, Te···O denotes the secondary bonds and Te· · · ·O suggests failing to meet the criteria for secondary bonds.

Supplementary Table 12. Inter-atomic distances, angles and solid angle of Te-O and Bi-O bonds in $Bi_2Te_4O_{11}$ for the average structural model at 600℃

Te1	Distance (Å)	Te1	Angle (°)	Solid Angle (°)
Te1-O4	1.878(9)			21.8
Te1-O5	1.961(10)			19.8
Te1-O6	1.914(11)			20.9
Te1···O8	2.781(9)	O4-Te1···O8	169.7(4)	11.7
Te1···O5	2.927(10)	O5-Te1···O5	173.6(4)	10
Te1· · · ·O6	3.013(11)	O6-Te1···O6	174.13(16)	4.3
Te1· · · ·O10	3.427(9)	O6-Te1· · · ·O10	138.2(4)	3.3
Te2	Distance (Å)	Te2	Angle (°)	Solid Angle (°)
Te2-O8	1.944(9)			21.4
Te2-O1	2.028(9)			18.6
Te2-O7	2.028(9)			17.9
Te2···O3	2.644(8)	O7-Te2···O3	165.2(4)	13.6
Te2···O1	2.792(9)	O8-Te2···O1	173.3(4)	10.8
Te2· · · ·O4	2.812(9)	O1-Te2· · · ·O4	141.6(4)	10.6
Te2· · · ·O6	3.431(10)	O1-Te2· · · ·O6	156.6(4)	2.7
Te2· · · ·O9	3.377(9)	O4-Te2· · · ·O9	156.7(3)	0.8
Te3	Distance (Å)	Te3	Angle (°)	Solid Angle (°)
Te3-O10	1.842(10)			20.9
Te3-O11	1.871(8)			19.8
Te3-O6	2.056(10)			19.8
Te3···O5	2.387(9)	O6-Te3···O5	177.7(4)	15.0
Te3···O10	2.814(10)	O10-Te3···O10	174.6(4)	10.4
Te3· · · ·O3	3.223(9)	O3-Te3· · · ·O10	130.2(4)	4.6
Te3· · · ·O11	3.541(8)	O11-Te3· · · ·O11	151.8(4)	2.7

Te4	Distance (Å)	Te4	Angle (°)	Solid Angle (°)
Te4-O3	1.857(8)			20.92
Te4-O9	1.994(9)			18.13
Te4-O2	1.887(9)			20.3
Te4···O10	2.557(9)	O2-Te4···O10	170.6(4)	13.69
Te4· · · ·O7	2.990(9)	O9-Te4···O7	140.8(4)	4.61
Te4···O2	3.012(9)	O3-Te4···O2	173.1(4)	9.3
Te4· · · ·O8	3.207(8)	O9-Te4· · · ·O8	155.6(4)	5.43
Bi1	Distance (Å)	Bi2	Distance (Å)	
Bi1-O2	2.205(8)	Bi2-O9	2.150(8)	
Bi1-O7	2.248(8)	Bi2-O1	2.248(8)	
Bi1-O7	2.246(7)	Bi2-O8	2.411(8)	
Bi1-O4	2.507(7)	Bi2-O11	2.469(7)	
Bi1-O3	2.505(8)	Bi2-O9	2.507(7)	
Bi1-O1	2.741(8)	Bi2-O2	2.823(7)	
Bi1-O11	2.667(8)	Bi2-O4	3.026(8)	
		Bi2-O5	2.932(7)	

* Te-O denotes the covalent bonds, Te···O denotes the secondary bonds and Te· · · ·O suggests failing to meet the criteria for secondary bonds.

Supplementary Table 13. EXAFS fitting parameters at the Bi L_3-edge for $Bi_2Te_2O_7$ and $Bi_2Te_4O_{11}$

Sample	Temperature(°C)	Path	*N*	*R* (Å)	σ^2 (Å^2)	ΔE_0 (eV)	*R*-factor (%)
$Bi_2Te_2O_7$	RT 25	Bi-O	3.5 ± 0.3	2.298 ± 0.009	0.0129	-2.1 ± 0.9	0.83
		Bi-O	1.1 ± 0.7	2.504 ± 0.014	± 0.0037		
		Bi-O	0.8 ± 1.0	2.729 ± 0.013			
$Bi_2Te_2O_7$	HT 650	Bi-O	2.5 ± 0.6	2.251 ± 0.014	0.0138	-5.3 ± 1.8	1.53
		Bi-O	0.5 ± 1.1	2.673 ± 0.019	± 0.0062		
		Bi-O	0.4 ± 1.2	3.046 ± 0.024			
$Bi_2Te_4O_{11}$	RT 25	Bi-O	5.1 ± 0.3	2.306 ± 0.009	0.0124	-2.9 ± 0.7	0.90
		Bi-O	1.3 ± 0.6	2.628 ± 0.014	± 0.0042		
		Bi-O	0.8 ± 0.4	2.794 ± 0.025			
$Bi_2Te_4O_{11}$	HT 600	Bi-O	1.7 ± 0.1	2.231 ± 0.013	0.0147	-7.1 ± 1.5	1.92
		Bi-O	1.4 ± 0.3	2.328 ± 0.019	± 0.0040		
		Bi-O	0.2 ± 1.1	2.485 ± 0.042			

* N: coordination numbers; *R*: bond distance; σ^2: Debye-Waller factors; ΔE_0: the inner potential correction; *R*-factor: the quality factor; S_0^2 were set as 0.902 for Bi-O, which was obtained from the experimental EXAFS fit of reference Bi powder by fixing CN as the known crystallographic value and was fixed to all the samples.

References

1. Alcock, N. W. Secondary bonding to nonmetallic elements. *Adv. Inorg. Chem. Radiochem.* **15**, 1–58 (1972).
2. Bleiholder, C., Werz, D. B., Köppel, H. & Gleiter, R. Theoretical investigations on chalcogen−chalcogen interactions: What makes these nonbonded interactions bonding? *J. Am. Chem. Soc.* **128**, 2666–2674 (2006).
3. Cozzolino, A. F., Elder, P. J. W. & Vargas-Baca, I. A survey of tellurium-centered secondary-bonding supramolecular synthons. *Coord. Chem. Rev.* **255**, 1426–1438 (2011).
4. Mohajeri, A., Pakiari, A. H. & Bagheri, N. Theoretical studies on the nature of bonding in σ-hole complexes. *Chem. Phys. Lett.* **467**, 393–397 (2009).
5. Si, M. K. & Ganguly, B. Computational evidence that hyperconjugative orbital interactions are responsible for the stability of intramolecular Te···O/Te···S non-covalent interactions and comparable to hydrogen bonds in quasi-cyclic systems. *New J. Chem.* **40**, 9132–9138 (2016).
6. Cozzolino, A. F., Vargas-Baca, I., Mansour, S. & Mahmoudkhani, A. H. The nature of the supramolecular association of 1,2,5-chalcogenadiazoles. *J. Am. Chem. Soc.* **127**, 3184–3190 (2005).
7. Chivers, T. & Laitinen, R. S. Tellurium: A maverick among the chalcogens. *Chem. Soc. Rev.* **44**, 1725–1739 (2015).
8. Kollman, P. A. & Allen, L. C. Theory of the hydrogen bond. *Chem. Rev.* **72**, 283–303 (1972).
9. Gilli, G. & Gilli, P. Towards an unified hydrogen-bond theory. *J. Mol. Struct.* **552**, 1–15 (2000).
10. Buckingham, A. D., Del Bene, J. E. & McDowell, S. A. C. The hydrogen bond. *Chem. Phys. Lett.* **463**, 1–10 (2008).
11. Silvi, B., Alikhani, E. & Ratajczak, H. Towards an unified chemical model of secondary bonding. *J. Mol. Model.* **26**, 62 (2020).
12. Steiner, T. The hydrogen bond in the solid state. *Angew. Chem. Int. Ed.* **41**, 48–76 (2002).
13. Grabowski, S. J. Theoretical studies of strong hydrogen bonds. *Annu. Rep. Sect. C Phys. Chem.* **102**, 131–165 (2006).
14. Zukerman-schpector, J. & Haiduc, I. Diorganotellurium(IV) dihalides and secondary bonding: Revisiting the coordination polyhedra. *Phosphorus Sulfur Silicon Relat. Elem.* **171**, 73–112 (2001).
15. Tuckerman, M., Laasonen, K., Sprik, M. & Parrinello, M. Ab initio molecular dynamics simulation of the solvation and transport of H_3O^+ and OH^- ions in water. *J. Phys. Chem.* **99**, 5749–5752 (1995).
16. Benoit, M., Marx, D. & Parrinello, M. Tunnelling and zero-point motion in high-pressure ice. *Nature* **392**, 258–261 (1998).
17. Spohr, E., Commer, P. & Kornyshev, A. A. Enhancing proton mobility in polymer electrolyte membranes: Lessons from molecular dynamics simulations. *J. Phys. Chem. B* **106**, 10560–10569 (2002).
18. Kühlbrandt, W. Bacteriorhodopsin — the movie. *Nature* **406**, 569–570 (2000).
19. Wu, X., Hong, J. J., Shin, W., Ma, L., Liu, T., Bi, X., *et al.* Diffusion-free Grotthuss topochemistry for high-rate and long-life proton batteries. *Nat. Energy* **4**, 123–130 (2019).
20. Sahoo, R., Mondal, S., Pal, S. C., Mukherjee, D. & Das, M. C. Covalent-organic frameworks (COFs) as proton conductors. *Adv. Energy Mater.* **11**, 2102300 (2021).
21. Chou, S.-C., Höss, P., Russ, P. L., Strobel, S. & Schleid, T. New crystal structures of rare-earth

metal(III) oxotellurates(IV) $RE_2Te_3O_9$: A1-type (RE=La, Ce) and A2-type (RE=Pr, Nd). *Z. Für Anorg. Allg. Chem.* **647**, 134–150 (2021).

23. Zemann, J. Zur stereochemie des Te(IV) gegenüber sauerstoff. *Monatshefte Für Chem. Chem. Mon.* **102**, 1209–1216 (1971).
23. Blatov, V. A. Voronoi–dirichlet polyhedra in crystal chemistry: Theory and applications. *Crystallogr. Rev.* **10**, 249–318 (2004).
24. Mekki, A., Khattak, G. D. & Wenger, L. E. Structural and magnetic properties of MoO_3–TeO_2 glasses. *J. Non-Cryst. Solids* **351**, 2493–2500 (2005).
25. Himei, Y., Osaka, A., Nanba, T. & Miura, Y. Coordination change of Te atoms in binary tellurite glasses. *J. Non-Cryst. Solids* **177**, 164–169 (1994).
26. Li, M., Pietrowski, M. J., De Souza, R. A., Zhang, H., Reaney, I. M., Cook, S. N., *et al.* A family of oxide ion conductors based on the ferroelectric perovskite $Na_{0.5}Bi_{0.5}TiO_3$. *Nat. Mater.* **13**, 31–35 (2014).
27. Islam, M. S. Ionic transport in ABO_3 perovskite oxides: A computer modelling tour. *J. Mater. Chem.* **10**, 1027–1038 (2000).
28. Aidhy, D. S., Sinnott, S. B., Wachsman, E. D. & Phillpot, S. R. Effect of ionic polarizability on oxygen diffusion in δ-Bi_2O_3 from atomistic simulation. *Ionics* **16**, 297–303 (2010).
29. Schütz, D., Deluca, M., Krauss, W., Feteira, A., Jackson, T. & Reichmann, K. Lone-pair-induced covalency as the cause of temperature- and field-induced instabilities in bismuth sodium titanate. *Adv. Funct. Mater.* **22**, 2285–2294 (2012).
30. Walsh, A., Watson, G. W., Payne, D. J., Edgell, R. G., Guo, J., Glans, P.-A., *et al.* Electronic structure of the α and δ phases of Bi_2O_3: A combined ab initio and x-ray spectroscopy study. *Phys. Rev. B* **73**, 235104 (2006).
31. Nguyen, T., Ishihara, T., Kilner, J. & Staykov, A. Effect of electronic interactions on oxide-ion mobility in solid electrolytes with a fluorite structure. *J. Phys. Chem. C* **127**, 22437–22446 (2023).
32. Lu, W., Gao, Z., Du, X., Tian, X., Wu, Q., Li, C., *et al.* Shedding light on the intrinsic characteristics of 3D distorted fluorite-type zirconium tellurite single crystals. *Inorg. Chem.* **58**, 7794–7802 (2019).
33. Földvári, I., Péter, Á., Szakács, O. & Munoz F, A. Improvement in quality and performance of photorefractive Bi_2TeO_5. *J. Cryst. Growth* **198–199**, 482–486 (1999).
34. Meier, S. F. & Schleid, T. Oxotellurate(IV) der lanthanide: I. Die isotype reihe $M_2Te_4O_{11}$ (M = La – Nd, Sm – Yb). *Z. Für Naturforschung B* **59**, 881–888 (2004).
35. Champarnaud-Mesjard, J.-C., Frit, B., Chagraoui, A. & Taïri, A. Crystal structure of a new cation-ordered fluorite-related phase: $Bi_2Te_2WO_{10}$. *Z. Für Anorg. Allg. Chem.* **622**, 1907–1912 (1996).
36. Hartmann, E. Electrical conductivity of Bi_2TeO_5 single crystals at high temperatures. *Cryst. Res. Technol.* **36**, 911–916 (2001).
37. Perdew, J. P., Ruzsinszky, A., Csonka, G. I., Vydrov, O. A., Scuseria, G. E., Constantin, L. A., *et al.* Restoring the density-gradient expansion for exchange in solids and surfaces. *Phys. Rev. Lett.* **100**, 136406 (2008).
38. Kühne, T. D., Iannuzzi, M., Del Ben, M., Rybkin, V. V., Seewald, P., Stein, F., *et al.* CP2K: An electronic structure and molecular dynamics software package - quickstep: Efficient and accurate electronic structure calculations. *J. Chem. Phys.* **152**, 194103 (2020).

39. Goedecker, S., Teter, M. & Hutter, J. Separable dual-space Gaussian pseudopotentials. *Phys. Rev. B* **54**, 1703–1710 (1996).
40. VandeVondele, J. & Hutter, J. Gaussian basis sets for accurate calculations on molecular systems in gas and condensed phases. *J. Chem. Phys.* **127**, 114105 (2007).
41. Togo, A. First-principles phonon calculations with phonopy and phono3py. *J. Phys. Soc. Jpn.* **92**, 012001 (2023).
42. Himei, Y., Miura, Y., Nanba, T. & Osaka, A. X-ray photoelectron spectroscopy of alkali tellurite glasses. *J. Non-Cryst. Solids* **211**, 64–71 (1997).
43. Ceriotti, M., Pietrucci, F. & Bernasconi, M. Ab initio study of the vibrational properties of crystalline TeO_2: The α, β, and γ. *Phys. Rev. B* **73**, 104304 (2006).
44. Bachvarova-Nedelcheva, A., Iordanova, R., Kostov, K. L., Ganev, V. & Yordanov, St. Synthesis, characterization and optical properties of non-traditional tellurite–selenite glasses. *Opt. Mater.* **36**, 1319–1328 (2014).
45. Zhang, G., Cai, L., Zhang, Y. & Wei, Y. Bi^{5+}, $Bi^{(3-x)+}$, and oxygen vacancy induced $BiOCl_xI_{1-x}$ solid solution toward promoting visible-light driven photocatalytic activity. *Chem. – Eur. J.* **24**, 7434–7444 (2018).
46. Heo, J., Lam, D., Sigel Jr., G. H., Mendoza, E. A. & Hensley, D. A. Spectroscopic analysis of the structure and properties of alkali tellurite glasses. *J. Am. Ceram. Soc.* **75**, 277–281 (1992).
47. Ou, G., Xu, Y., Wen, B., Lin, R., Ge, B., Tang, Y., *et al.* Tuning defects in oxides at room temperature by lithium reduction. *Nat. Commun.* **9**, 1302 (2018).
48. An, P., Hong, C., Zhang, J., Xu, W. & Hu, T. A facile heating cell for in situ transmittance and fluorescence X-ray absorption spectroscopy investigations. *J. Synchrotron Radiat.* **21**, 165–169 (2014).
49. Dennington, R., Keith, T. A. & Millam, J. M. GaussView Version 6. (2019).
50. Arnaudov, M., Dimitrov, V., Dimitriev, Y. & Markova, L. Infrared-spectral investigation of tellurites. *Mater. Res. Bull.* **17**, 1121–1129 (1982).
